# Cryomicroscopy reveals the structural basis for a flexible hinge motion in the immunoglobulin M pentamer

**DOI:** 10.1038/s41467-022-34090-2

**Published:** 2022-10-23

**Authors:** Qu Chen, Rajesh Menon, Lesley J. Calder, Pavel Tolar, Peter B. Rosenthal

**Affiliations:** 1 Structural Biology Science Technology Platform, The Francis Crick Institute, 1 Midland Road, London, NW1 1AT UK; 2 Immune Receptor Activation Laboratory, The Francis Crick Institute, 1 Midland Road, London, NW1 1AT UK; 3 Structural Biology of Cells and Viruses Laboratory, The Francis Crick Institute, 1 Midland Road, London, NW1 1AT UK; 4 Institute of Immunity and Transplantation, University College London, Rowland Hill Street, London, NW3 2PP UK

**Keywords:** Cryoelectron microscopy, Cryoelectron microscopy, Antibodies

## Abstract

Immunoglobulin M (IgM) is the most ancient of the five isotypes of immunoglobulin (Ig) molecules and serves as the first line of defence against pathogens. Here, we use cryo-EM to image the structure of the human full-length IgM pentamer, revealing antigen binding domains flexibly attached to the asymmetric and rigid core formed by the Cμ4 and Cμ3 constant regions and the J-chain. A hinge is located at the Cμ3/Cμ2 domain interface, allowing Fabs and Cμ2 to pivot as a unit both in-plane and out-of-plane. This motion is different from that observed in IgG and IgA, where the two Fab arms are able to swing independently. A biased orientation of one pair of Fab arms results from asymmetry in the constant domain (Cμ3) at the IgM subunit interacting most extensively with the J-chain. This may influence the multi-valent binding to surface-associated antigens and complement pathway activation. By comparison, the structure of the Fc fragment in the IgM monomer is similar to that of the pentamer, but is more dynamic in the Cμ4 domain.

## Abstract (web summary)

Immunoglobulin M (IgM) is the Ig isotype that serves as the first line of host defence during infection. Here, the authors image the full-length IgM pentamer by cryo-EM, revealing the structure and hinge motion of the antigen binding domains.

## Introduction

Natural immunoglobulin M (IgM) assembles into monomers (composed of two heavy and two light chains), pentamers and hexamers. Monomeric IgM serves as a membrane-associated antigen-recognition receptor on the surface of B lymphocytes^1^. Pentameric IgM is secreted into serum, or transported across epithelial cells by the polymeric immunoglobulin receptor (pIgR)^2,3^. IgM hexamers have also been found in the serum, but account for less than 5% of total IgM^4^. An IgM pentamer is over 900 kDa, consisting of ten heavy (μ) chains, each of which has a C-terminal tailpiece which holds them together, ten light (λ or κ) chains and a joining (J) chain, a 15 kDa polypeptide ﻿with no homology to other proteins, which sterically interrupts the addition of a sixth heavy crystal fragment (Fcμ) unit^5–7^. As the very first antibody produced after pathogen invasion, the multivalent nature of polymeric IgM molecules gives them remarkable ability to ﻿agglutinate particulate antigens in serum^8–10^.

Efforts have been made for decades to study the structure of IgM but have been hindered by its size and flexibility. Individual Cμ4, Cμ3, Cμ2 and Fab domains have been resolved by X-ray crystallography and NMR^11–17^, complexes of IgM with complement components C1 and C4b have been studied at low resolution by electron cryotomography^18^, and two recent publications revealed the atomic structure of the asymmetric pentameric Cμ4-Cμ3 core by single-particle cryo-EM^19,20^. Nevertheless, important questions such as how the peripheral domains are positioned and move with respect to the Fcμ core remain to be answered, which can only be achieved by imaging the structure of FL-IgM as a whole. Traditionally, structures of the hinge regions of immunoglobulin molecules have been identified by enzymatic fragmentations and sequence homology, according to which IgG and IgA are classified into several subclasses^21–23^. However, little sequence similarity has been found at the hinge-equivalent region of IgM owing to the substitution of an additional Fc domain Cμ2, which has led to the conclusion that IgM lacks a hinge region^24,25^. This does not necessarily imply that IgM lacks flexibility at this region, and the two potential origins of flexibility are the interdomain regions between Cμ1 and Cμ2, and between Cμ2 and Cμ3. Evidence of flexibility between the Fc and Fab fragments of IgM have been shown in several studies, where projections of IgM alone or ligand-bound forms were captured in negative stain EM images^7,26,27^. However, where exactly the flexibility comes from, whether at the N- or C-terminus of the Cμ2 domain or both is still ambiguous.

In this study, we have addressed these questions by analysing the 3D structure of human FL-IgM using a single particle cryo-EM approach, placing the emphasis on understanding the hinge structure and flexibility of IgM. The pentameric core serves as a high molecular weight scaffold to study the structure of the flexibly-attached Fab regions. We resolve an F(ab’)_2_ at one position of the pentamer, which is stabilised with respect to the core, and interpret disordered density distributions at other Fab positions in terms of a hinge-equivalent motion of IgM. In comparison, we study the structure of the IgM Fc monomer by both cryo-EM and time-resolved single-molecule fluorescence resonance energy transfer (smFRET). We show that while the structure of the monomer is similar to that of the pentameric core, its Cμ4 domains undergo dynamic separation, which may be relevant to IgM function as the membrane-bound B cell receptor.

## Results and discussion

### Cryo-EM Imaging of full-length IgM Pentamers

**Fig. 1:**
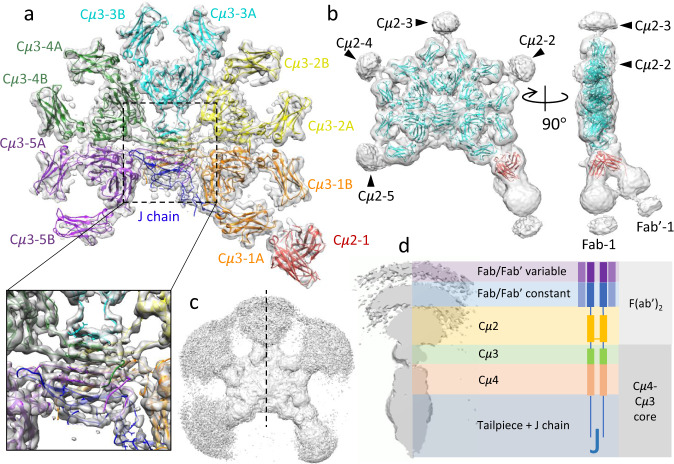
The cryo-EM structure of FL-IgM. **a** Sharpened cryo-EM map of FL-IgM showing the high-resolution core structure (threshold 0.6). Global resolution 4.3 Å. The model docked in Cμ4-Cμ3 areas is IgM core model (pdb id 6KXS, with the secretory component (SC) domain removed) with a unique colour assigned for each subunit (subunit 1, orange; subunit 2, yellow; subunit 3, cyan; subunit 4, green; subunit 5, purple). The model docked in the Cμ2 domain of subunit 1 is the X-ray model of mouse Cμ2 dimer (red, pdb id 4JVU). The inset is the zoom-in view of the tailpiece and J-chain region, where individual beta-strands are resolved (local resolution 3.5 Å, see maps for tailpiece and J-chain segments in Supplementary Fig. 2). The resolution of the map decreases along the radial directions from the centre, with 4-5 Å resolution at the Cμ4 domains and 5.5-7.5 Å at the Cμ3 domains for all the ten copies of the IgM Fc core (except for Cμ3-5B, which is about 1 Å resolution lower than the other Cμ3s). Cμ2 in subunit 1 reaches an intermediate-resolution level (6–8 Å). The map coloured with local resolutions is shown in Supplementary Fig. 1g. **b** Unsharpened cryo-EM map of FL-IgM with the threshold set at 0.18 with the same models docked as in (**a**) (cyan, 6KXS; red, Cμ2). The densities corresponding to Cμ2 domains in subunit 2–5 are indicated by black arrowheads. Densities of the two Fab arms are identifiable in subunit 1 (Fab-1 and Fab’−1). **c** The same map as (**b**) at threshold level 0.025. **d** Half of the cross-section of the map in (**c**) at the black dotted line in (**c**), with a schematic of FL-IgM (one subunit) as well as notation given for each component domain.

Maps of full-length IgM (FL-IgM) obtained by cryo-EM (workflow in Supplementary Fig. 1) show a rigidly assembled core and flexible Fabs at peripheral regions of each subunit. The core, including Cμ3, Cμ4, tailpiece and J-chain segments (Fig. 1a), is highly similar to the previously published cryo-EM structure of the IgM core (Supplementary Fig. 1d and Supplementary Fig. 2)^19^, despite the absence of the secretory component (SC) chain in our sample, indicating that the core is largely stabilised by the tailpiece and J-chain. Similarly, dimeric IgA with and without SC have been found to have relatively small structural differences^28^.

The local resolution decreases radially within the cryo-EM map, indicating that the flexibility increases from the core to peripheral regions of IgM molecule. As with the previously published core structure, our maps show that Cμ3 has higher degrees of freedom than Cμ4^19,20^, and our map further reveals the highly flexible Cμ2 and Fabs. To our surprise, not all Cμ2s are equally flexible: the Cμ2-1 domain in subunit 1 has very limited flexibility, confirmed by the additional densities extending from the Cμ3-1A domain in Fig. 1a. The two subunits within the Cμ2-1 dimer are clearly identifiable in the map, which is not the case for the other four Cμ2s viewed at the same threshold level.

Densities at the flexible domains are visible at a lower map contour level (Fig. 1b). Ellipsoid-shaped densities corresponding to the Cμ2 domains in subunits 2–5 are identifiable (black arrow heads in Fig. 1b), as well as the two Fab arms in subunit 1 (Fab-1/Fab’−1). The map densities of these areas are spherical shapes, indicating that the densities are the sum of several continuous conformations. The densities of Fab/Fab’ in subunits 2-5 appear when further lowering the threshold level of the map (Fig. 1c), revealing more conformational flexibility. The densities can be regarded as 3-D probability distributions for the Fab positions. Figure 1d is a cross section through the middle of the map (black dotted line in Fig. 1c) revealing features from the core to the Fab.

**Fig. 2:**
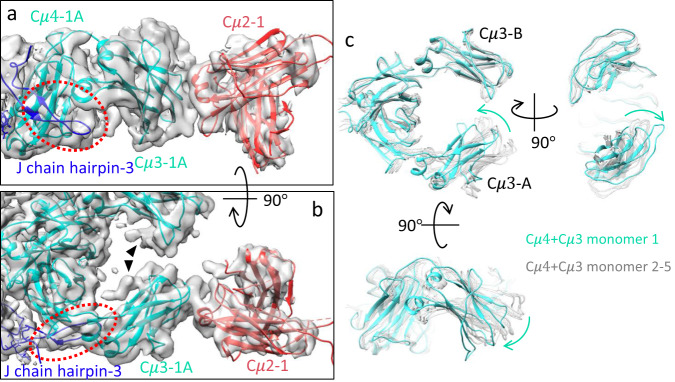
Stabilisation of Cμ2 domain in subunit 1. **a**, **b** Zoomed-in view at threshold 0.6 of Cμ2-1 (red, pdb id 4JVU), Cμ3-1 A - Cμ4-1 A (cyan, pdb id 6KXS) and hairpin-3 of J-chain (dark blue) showing the relationships between the domains. The red dotted ellipse highlighting the interacting sections between J-chain and Cμ3-1 A. The black arrowheads in (**b**) point at the densities corresponding to N-linked glycosylation at N402 and N395 (densities of glycosylation are visible at all the other Cμ3 densities in Fig. 1a). **c** Overlay of the Cμ4-Cμ3 models of subunit 1 (cyan) and the rest of the subunits (subunit 2-5, light grey). The cyan arrows indicate the relative displacement of Cμ3-1 A.

Within the IgM pentamer, the unique rigidity of F(ab’)_2_ in subunit 1 is correlated to the interaction between J-chain and Cμ3-1 A domain. Previously, Li et al.^19^, observed that the C-terminal loop in the J-chain (hairpin-3 in Fig. 2a, b) extends towards and forms hydrophobic contacts with Cμ3-1 A domain. In addition, there is no neighbouring Cμ3 domain to form an interchain disulfide bond at C414, leading to a distinct orientation of Cμ3-1 A compared to the other interlocking Cμ3 domains. Superimposing Cμ3 and Cμ4 domains in subunits 1 and 2–5 (Fig. 2c) reveals that Cμ3-1 A (cyan) is bent both more inward and out-of-plane compared to the other Cμ3-A domains.

In contrast to the highly flexible Cμ2 domains which are symmetrically associated with the two adjacent Cμ3 domains in subunits 2-5 (Fig. 1b), Cμ2-1 is docked and leaning towards Cμ3-1 A, positioning the short ${{{{{\rm{\alpha }}}}}}$-helix (S308-N314) of Cμ2 near the tip of Cμ3-1 A (T429-P436) which may contribute to the stabilisation of Cμ2-1 (Fig. 2a–b), though higher resolution data are required to identify detailed side chain interactions. In the model, the straight distances from the C-termini of Cμ2-1 (C337) to the N-termini of Cμ3-1 A and Cμ3-1B (I345) are 21 Å and 19 Å respectively (Supplementary Fig. 3a), both of which are much shorter than and therefore compatible with the fully extended linker lengths of eight residues at 3.5 Å per residue. The same stabilization of Cμ2 and Fab does not occur at subunit 5 on the other side of the molecule, which may be a consequence of the weaker interaction between the J-chain and Cμ3-5B^29^.

### Structure/conformations of antigen binding F(ab’)_2_ in subunits

**Fig. 3:**
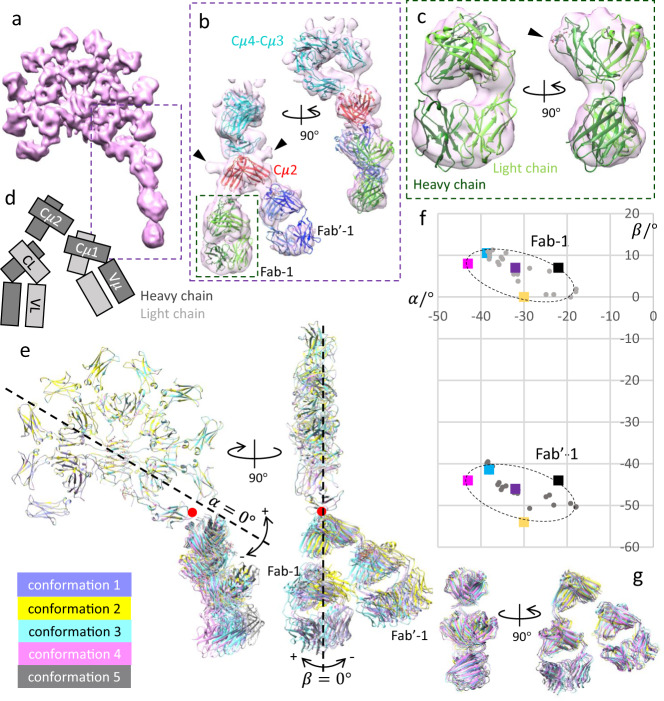
Structure of F(ab’)_2_ in subunit 1 and its flexibility. **a** One of five conformations (conformation 4) classified from 3-D variability analysis (3DVA). Threshold of the map is 2.4. **b** Map obtained by focused-refinement of the conformation 4 at subunit 1 (dotted box in (**a**)). B factor used for map sharpening is −400 Å^2^ and the threshold of the map is 2. The two black arrowheads point at the N-linked glycosylation at N332 in Cμ2 dimer. **c** Zoomed-in view of Fab-1 (green dotted box in (**b**)), with location of oligosaccharide at N165 on heavy chain indicated by the black arrowhead. Models docked in (**b**, **c**) are pdb id 6KXS (cyan, subunit 1 only), pdb id 4JVU (red) and two crystal models of IgM Fab (green and blue, pdb id 2AGJ) for Fab-1 and Fab’−1. The heavy chains are dark green/blue and light chains are light green/blue. The maps for the other four conformations and corresponding focused refined maps are in Supplementary Fig. 5. **d** Schematic of the heavy and light chain arrangement in F(ab’)_2_. **e** Overlay of the models of five maps with different F(ab’)_2_ conformations aligned at their common Cμ4-Cμ3 core region. The in-plane (${{{{{\rm{\alpha }}}}}}$) and out-of-plane (${{{{{\rm{\beta }}}}}}$) and the positive/negative axes of F(ab’)_2_ are defined. The orientation of each Fab/Fab’ arm is defined as the direction from the C-terminus of Cμ2-1 to the centre of the constant domain ${{{{{\rm{\alpha }}}}}}=0^\circ$ is defined as the two-fold symmetry axis of Cμ4-1 A/1B dimer, and ${{{{{\rm{\beta }}}}}}=0^\circ$ is the platform formed by Cμ4-Cμ3 core (black dotted lines). ${{{{{\rm{\alpha }}}}}}$ and ${{{{{\rm{\beta }}}}}}$ are the relative angles between Fab/Fab’ and ${{{{{\rm{\alpha }}}}}}=0^\circ$/${{{{{\rm{\beta }}}}}}=0^\circ$. **f** Summary of ${{{{{\rm{\alpha }}}}}}$ and ${{{{{\rm{\beta }}}}}}$ in the five conformations. The datapoints are the same colours as the maps in (**e**). The grey datapoints are the Fab/Fab’ values measured from the 20 frames in the volume series calculated by 3DVA showing the continuous flexibility of F(ab’)_2_ in subunit 1 (Supplementary Movie 1 and Supplementary Movie 2). **g** Superimposition of the F(ab’)_2_ structures in all five conformations.

In order to resolve distinct conformations within our cryo-EM map, we conducted 3D variability analysis^30^ which reveals the F(ab’)_2_, which includes both Fabs and Cμ2 domains, moves as a rigid body and is able to pivot at the Cμ2/Cμ3 interface. Five conformations have been classified from the full dataset (Supplementary Fig. 4), with conformation 4 (pink) displayed in Fig. 3a (the other four maps in Supplementary Fig. 5). While the Cμ4-Cμ3 core structures look almost identical among the five classes, they are distinguishable by different orientations of F(ab’)_2_ in subunit 1. Focused refinement on subunit 1 (dotted box region in Fig. 3a) better resolves the structure of Fab domains, which are shown in Fig. 3b, c (the other four conformations in Supplementary Fig. 5). The two subunits of the constant domain of the Fab (Cμ1 and CL), as well as the elbows connecting the constant and variable domains, fit well at the domain level between the map and the crystal model pdb id, 2AGJ^31^) for all the Fab domains (except for Fab in conformation 2 in Supplementary Fig. 5b, where the density of the variable domain is weaker). Figure 3d illustrates the arrangement of the heavy chains and light chains of F(ab’)_2_ adopted by the five conformations, where the two inner chains are light chains, and the outer chains, more proximal to Cμ2, are heavy chains. This arrangement has slightly better agreement than the configuration with each Fab rotated 180° about its approximate two-fold axis (i.e., heavy chain inside, light chain outside), in fitting the oligosaccharide at N165 (black arrowhead in Fig. 3c). The defined conformation of Fab/Fab’ suggests a stabilised association between Cμ2 and the Fab constant domain.

Superimposing the models for five configurations aligned at the Cμ4-Cμ3 core region shows the different orientations of F(ab’)_2_ (Fig. 3e), revealing that the pivot point for F(ab’)_2_ (red dots in Fig. 3e) is at the Cμ2/Cμ3 interface. Supplementary Movie 1 (front view) and Supplementary Movie 2 (side view) display the flexibility as continuous movements, where F(ab’)_2_ pivots at the Cμ2/Cμ3 interface along both parallel and normal directions to the Cμ4-Cμ3 core. No translational movement or rotation about the two-fold symmetry axis of F(ab’)_2_ is present. The orientations of F(ab’)_2_ are summarised in Fig. 3f (definitions and measurements are described in the figure legend). The range of in-plane angle ${{{{{\rm{\alpha }}}}}}$ is between $-20^\circ$ to $-45^\circ$ (same as measured for either Fab and Fab’), and the range of out-of-plane angle ${{{{{\rm{\beta }}}}}}$ is from $-10^\circ$ to $0^\circ$ for Fab-1, and $-40^\circ$ to $-55^\circ$ for Fab’−1. The relative angle between the two Fab arms is a constant about $50^\circ$, and superimposing the five F(ab’)_2_ structures shows that they share an identical conformation (Fig. 3g) and indicate a rigid F(ab’)_2_ structure in subunit 1 of the IgM molecule.

The rigidity of IgM F(ab’)_2_ is supported by a previous study, where a pair-wise surface binding behaviour was observed in an IgM-surface antigen complex with complement components studied by cryotomography, i.e., both Fab and Fab’ are facing towards the binding surface in contrast to only one in IgG (where the other Fab arm is dangling)^18,32^. This behaviour has been observed in all five subunits in the ﻿surface-bound IgM and the relative angles between the two Fab arms in the surface-bound IgM are also about $50^\circ$ to $55^\circ$^18^. This supports the idea that the rigidity of F(ab’)_2_ is intrinsic and universal in all subunits of the IgM molecule. The potential elbow motion between the constant and variable domains in the Fab is small compared to the observed F(ab’)_2_ flexibility but nevertheless important for antigen binding mechanisms for immunoglobulins^33^. Another phenomenon worth noting is that pentameric IgM is asymmetric with a biased Fab-1/Fab’−1 orientation towards the $-{{{{{\rm{\beta }}}}}}$ side, which is also the side identified by J-chain hairpin-3. The $120^\circ$ bending of F(ab’)_2_ observed in the surface-bound IgM^18^, much larger than the $55^\circ$ (Fig. 3f) in the free-form IgM, indicates that F(ab’)_2_ of subunit 1 can be highly strained when binding to surface antigens. It cannot be achieved by simply extending the pivot motion to 120° because the Cμ2-1 and Cμ3-1 A domain would clash, instead requiring an extension of the linker between Cμ2 and Cμ3 domain to accommodate the extreme bending angle of F(ab’)_2_. The intrinsic asymmetry of the free IgM suggests that it is more energetically favourable for IgM to engage surface antigens using the $-{{{{{\rm{\beta }}}}}}$ side, predisposing the $+{{{{{\rm{\beta }}}}}}$ side to engage with the C1q molecule of complement^34^. The C1q binding site on Cμ3-1 may be identified by superimposing the IgM conformations and IgG-Fc/C1q complex model (pdb id 6FCZ^32^), which reveals steric clashes between Cμ2-1 and C1q. Therefore, the C1q binding site on Cμ3-1 may be inaccessible for C1q binding until Cμ2-1 is displaced, such as when IgM binds to surface antigens.

IgM is widely described as lacking a hinge region, which is replaced by a whole Fc domain Cμ2^34^. A typical hinge in an immunoglobulin molecule such as IgG or IgA usually consists of three parts – an upper hinge at the C_H_1 C-terminus, a core hinge composed of multiple prolines and cysteines, and a lower hinge at the N-terminus of C_H_2 domain. The lower hinge allows the movement of the two Fab arms relative to the Fc domains, while the upper hinge provides independent freedom for each Fab, as demonstrated in previously published studies of IgG and IgA^28,35,36^. The hinge-equivalent region in IgM is Cμ2 as well as its two junctions at the N- and C-termini. Cμ2 itself is a rigid dimer linked by a disulfide bond, which spatially separates Fabs from the Cμ4-Cμ3 core as a core hinge does in IgGs and IgAs^21^. The N-termini of Cμ2 with the proline-rich linker region seem not to be able to provide motions (very little if any) for each Fab arm, as suggested by the observation of the rigid V-shaped F(ab’)_2_ configuration in subunit 1 of IgM. The C-termini of Cμ2 are flexible which resembles a classic lower hinge^37^, allowing the F(ab’)_2_ to move relative to the core. However, the range of freedom is limited by its short length which explains why the flexibility of F(ab’)_2_ in subunit 1 is analogous to pivoting instead of more complicated behaviours.

**Fig. 4:**
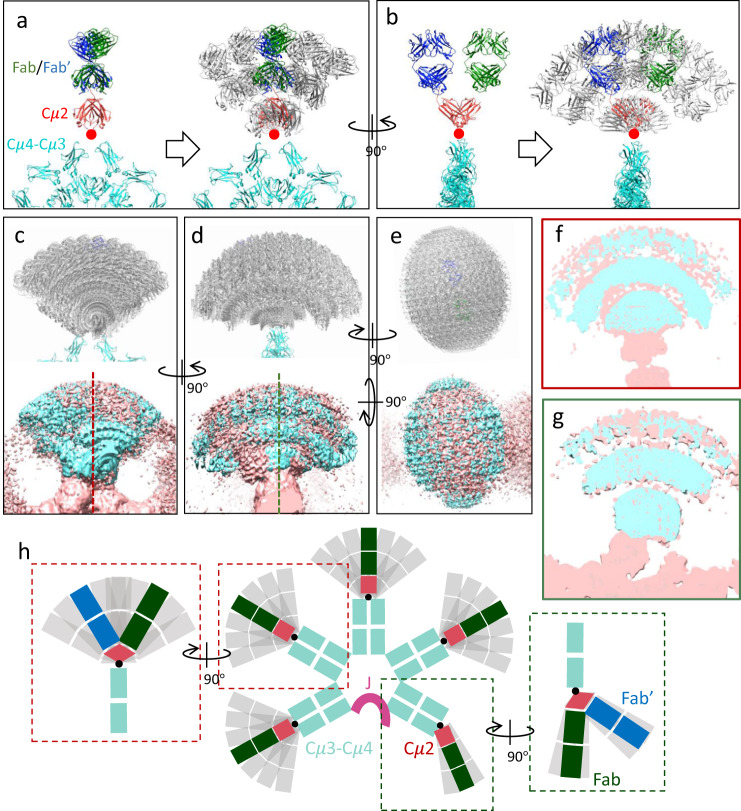
Model-based interpretation of the density of F(ab’)_2_ in subunit 2-5 based on the structure and flexibility of F(ab’)_2_ in subunit 1. **a** F(ab’)_2_ model rigidly translated from subunit 1 to subunit 4 (left panel), and in-plane pivoting of F(ab’)_2_ at the Cμ2/Cμ3 interface (right panel). **b** Left panel, side view of the left panel in (**a**). Right panel, out-of-plane pivoting of F(ab’)_2_ at Cμ2/Cμ3 interface. Red dots in (**a**, **b**) are the pivot point. **c**-**e** Upper panel, array of F(ab’)_2_ models with the in-plane and out-of-plane ranges of Cμ2 rotation at both $\pm 50^\circ$ with $10^\circ$ interval. The actual in-plane and out-of-plane directions are slightly tilted to match the tilted two-fold axes of the F(ab’)_2_ density (shown in Supplementary Fig. 6a–c). Lower panels, overlay of the experimental (pink) and simulated map (cyan) of F(ab’)_2_. Threshold level of the map is 0.025. (**f**, **g**) Cross-sections of the overlayed maps indicated by the dotted lines in (**c**) and (**d**). **h** Illustration of the overall structure of FL-IgM showing the two levels of flexibilities of F(ab’)_2_ among the five subunits.

The reconstructed map is the convolution of the map density of the molecule with the functions that describe the motions, so knowing the structure of F(ab’)_2_ and the pivot motion in subunit 1 explains the density distributions in subunit 2-5. Pivoting the Cμ2 model by $\pm 50^\circ$ along both the in-plane and out-of-plane directions gives a good agreement with the shape of the map density (Supplementary Fig. 6a and Supplementary Fig. 6b), and the two symmetry axes of the density are tilted about $10^\circ$ anti-clockwise from the Cμ4-Cμ3 platform (Supplementary Fig. 6c). When the pivoting motion is applied to the whole F(ab’)_2_, one can see a good agreement between simulated data and experimental data (Fig. 4a to 4g, Supplementary Fig. 7), which implies that the motion of F(ab’)_2_ we propose here can explain the cryo-EM map densities reasonably well and F(ab’)_2_ can indeed bend to much larger angles when not stabilised by the J-chain. Because of the $50^\circ$ splaying of the two Fab arms in the out-of-plane direction (Fig. 3e, f), the map density range along the out-of-plane direction appears larger by about $25^\circ$, roughly half the angle between the two Fab arms. This is confirmed by quantitatively analysing the map densities (method and calculations in ‘Quantification of the range of Fab motion in subunit 2-5’ in Methods and Supplementary Fig. 8 and Supplementary Fig. 9). For all four subunits 2-5, the map density range along the in-plane direction is in the range of $\pm 50^\circ$−6$0^\circ$ (Supplementary Fig. 9c and Supplementary Fig. 9e) while the range along the out-of-plane direction is about $\pm 70^\circ$-$80^\circ$ (Supplementary Fig. 9d and Supplementary Fig. 9f). This agrees well with the $\pm 50^\circ$ motion range along both in-plane and out-of-plane directions estimated from the model-based interpretation. When binding to surface antigens, an additional 40°–50° bending is required to accommodate the ‘staple-form’ IgM as well as expose the C1q binding sites^18^, possibly accompanied by some degree of linker extension similarly to subunit 1.

### Structure and dynamics of the IgM monomer

**Fig. 5:**
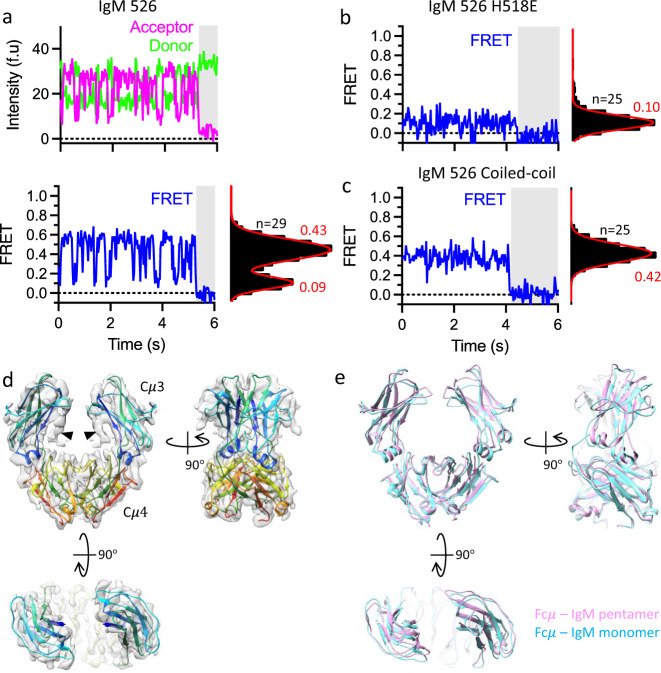
Dynamics of the IgM monomer based on smFRET and cryo-EM structure comparisons. **a** FRET donor (green) and acceptor (magenta) fluorescence intensity traces and the calculated FRET efficiency (blue) from a single molecule of a monomeric IgM-Fc construct labelled with the fluorophores at position 526 in the EF helix of Cμ4. The shaded area indicates when the acceptor molecule is photobleached. The histogram along the right FRET axis shows the distribution of the single-molecule FRET values for the *n* molecules measured. **b**, **c** Examples of single-molecule FRET traces and the respective distribution of FRET values for monomeric IgM-Fc constructs labelled at position 526 and containing either a mutation H518, which disrupts Cμ4 dimerization (**b**) or C-terminal coiled-coil, which forms a stable dimer (**c**). **d** Cryo-EM structure of IgM-Fc monomer with C-terminal coiled-coil showing the two-fold symmetrical arrangement at Cμ4 and Cμ3 regions (threshold 0.47). Black arrowheads indicate the density corresponding to the N-linked glycosylation at N395. **e** Superimposing the models of monomeric and a pentameric subunit (subunit 4 in pdb 6KXS) IgM showing similarity of the two structures, with slight variations at the orientations of Cμ3 domains. Source data are provided as a Source Data file.

We have also studied the IgM-Fc monomer (defined as Cμ4-Cμ3-Cμ2 dimer) by both smFRET and cryo-EM for comparison to Fc in the pentamer. Two potential Cμ4 arrangements of the IgM-Fc monomer have been reported: one is the ‘parallel’ conformation proposed by ﻿Müller et al (pdb id 4JVW)^11^, while the other is ‘anti-parallel’, which resembles the subunits in the IgM pentamer and the C-terminal domains of other Ig molecules such as IgG^38^, IgA^39^ and IgE^40^ (Supplementary Fig. 10a–c). Intriguingly, our time-resolved smFRET (Supplementary Fig. 10d, e) at two different labelling positions (Supplementary Fig. 10a, b) showed that the Cμ4 dimer in the IgM monomer is dynamic, yielding two discrete FRET states, which interchange several times per second (Fig. 5a, and Supplementary Fig. 11a). A comparison of the experimentally measured distances of the two labelled residues to the predicted distances calculated from the ‘parallel’ and ‘anti-parallel’ conformations (Supplementary Fig. 10a, b, Supplementary Table 1) reveals that the high FRET states (‘closed’ conformation) agree with the ‘anti-parallel’ conformation found in pentameric IgM, while the low FRET states (‘open’ conformation) do not align with either of the conformations. In contrast, the IgG Fc produced only one FRET state, which agreed with the distances calculated from the crystal structure (Supplementary Fig. 10c, Supplementary Fig. 11b, c, and Supplementary Table 1). This is consistent with C${{{{{\rm{\gamma }}}}}}$3 forming a stable dimer^41^, as is the case for C${{{{{\rm{\epsilon }}}}}}$4^42^, but unlike Cμ4. We then modified the Cμ4-Cμ4 interactions and captured the ‘closed’ and ‘open’ conformations of IgM Fc separately. Mutation of the histidine at the centre of the Cμ4 dimer interface (H518E) disrupting dimerization produced a single low FRET state (‘open’ conformation, Fig. 5b), whereas stabilisation of the C-terminus by a coiled-coil domain produced a single FRET state corresponding to the high FRET state of the wild-type construct (‘closed’ conformation, Fig. 5c).

This observation indicates that the Cμ4 dimer in the IgM monomer is inherently dynamic and is stabilised by external interactions. In the IgM pentamer, it is achieved by the tailpieces^43^, neighbouring IgM subunits and the J-chain. For the monomeric IgM expressed on membrane, given that the stalk that connects the IgM monomer to the cell membrane lacks a stabilising disulfide bond that is present in IgG, IgA and IgE, stability can be potentially provided by the transmembrane helices or by interactions with the Igα and Igβ subunits. Therefore, the unique dynamics at the IgM Cμ4 domains may play a role in isotype-specific assembly of the membrane IgM into the B cell receptor complex or its function after antigen binding.

The cryo-EM structure of IgM-Fc stabilised by the coiled-coil domain further confirms the resemblance between the ‘closed’ conformation of the IgM-Fc monomer to the subunits among subunit 2-5 in the IgM pentamer (workflow in Supplementary Fig. 12). Figure 5d illustrates the IgM-Fc model aligned with the cryo-EM map and superimposing the Cμ3-Cμ4 backbone models of the Fcμ monomer and subunit 4 of the IgM pentamer shows a good match at Cμ4 dimer with RMSD = 0.72 Å, whereas subtle mismatch occurs at Cμ3 domains, where the Cμ3 in monomeric Fcμ is slightly closer to Cμ4 (Fig. 5e). The differences in the Cμ3 domains are in line with a stabilising effect of the interdomain disulfide bond at C414 in the IgM pentamer. The symmetrical structure of the IgM-Fc monomer also supports the idea that the asymmetry observed at subunit 1 in the IgM pentamer is attributed to the interaction with the J-chain.

By cryo-EM imaging, we have shown an asymmetric and flexible full-length IgM pentamer. The features of the structure are summarised in Fig. 4h. Our results show that the IgM core, including Cμ4, Cμ3 and J-chain, adopts a structure superimposable with that previously described^19,20^ also in the absence of the secretory component, consistent with the role of the secretory component in transport rather than signalling. We have found different flexibilities at the Fab domains compared to IgGs and IgAs, owing to its unique hinge-equivalent structure at the interface between the Cμ3 and Cμ2 domains. Our understanding of IgM motions may be applicable to IgE, which lacks the hinge region typical of IgG and IgA, but likely contains a similar hinge-equivalent region to IgM based on sequence comparison. Previous analysis of IgE structure suggests that IgE C${{{{{\rm{\epsilon }}}}}}$2 can undergo a similar motion as we have observed for IgM. A crystal structure of IgE-Fc shows a C${{{{{\rm{\epsilon }}}}}}$2 domain acutely bent to one side of the Fc${{{{{\rm{\epsilon }}}}}}$ interacting with C${{{{{\rm{\epsilon }}}}}}$3-C${{{{{\rm{\epsilon }}}}}}$4^40^, whereas a symmetric complex of IgE-Fc with antibody fragments stabilises C${{{{{\rm{\epsilon }}}}}}$2 in an extended position^44^. A cryo-EM structure of an IgE:FcεRI complex with antibody fragments also shows C${{{{{\rm{\epsilon }}}}}}$2 can adopt an extended position^45^.

The observed asymmetry of the IgM pentamer originates from the J-chain, which extends to and interacts with the Cμ3-1 A domain, leading to the stabilisation of Cμ2 and Fab domains. The complement cascade starts from concerted antigen engagement by IgM which exposes C1q binding sites on Cμ3 domains. IgM F(ab’)_2_ is likely to be strained during multivalent binding of surface antigens. Interestingly, the IgM hexamer has been reported to be at least several-fold more potent ﻿in deposition of complement than the IgM pentamer^4,46,47^. Because the difference is large and found to be more pronounced at low antigen concentrations^46^, it is likely the result of the way the Fabs can interact with antigen and expose C1q binding sites. The asymmetric behaviour of F(ab’)_2_ of subunit 1, which is indirectly stabilised by the J-chain and blocks the C1q binding site, could contribute to this difference in fully symmetric complement deposition from the IgM hexamer without the J-chain^18^. This suggests an important effect for the asymmetric behaviour of the Fabs in complement activation.

During the revision of this work, IgM-BCR structures were published by two independent groups^48,49^. Though the Fab regions display some flexibility, these structures show the Cμ2 and Fab domains in similar positions to those of the stabilised Cμ2 and Fab in the IgM pentamer subunit 1 (Supplementary Fig. 13a). It is striking that in both cases the stabilisation of Cμ2 results from a similar, asymmetric Cμ3 Fc region (Supplementary Fig. 13b), where one Cμ3 domain is displaced compared to the symmetric subunits of the IgM pentamer or the symmetric IgM Fc monomer (Supplementary Fig. 13c, d). While in the case of pentameric IgM this asymmetry may be attributed to interactions with the J-chain, in the IgM-BCR the Cμ3 asymmetry arises from the interaction of one Cμ3 domain with the Igα chain. It is reasonable to speculate that in the BCR, Cμ3 asymmetry may couple the Fab domain to membrane components involved in B-cell signalling and that this may involve mechanisms that are conserved in secreted pentameric IgM.

We have also shown that the Cμ4 domains in the IgM monomer are inherently dynamic, but are stabilised by a coiled-coil which may mimic the transmembrane segments. Coiled-coil interactions have now been visualised in the transmembrane region of the IgM-BCR structures. Furthermore, the topological complexity of the membrane proximal region may be consistent with a requirement for co-assembly of IgM and Igαβ to form the IgM-BCR^48,49^. Given that the membrane IgM is unique in lacking a disulphide bond in the stalk, a dynamic separation of the Cμ4 domains may help regulate the assembly of IgM with Igαβ to form the IgM-BCR complex in an isotype-specific manner, or regulate activation of the IgM BCR after antigen binding.

Overall, our findings on the structure and flexibility of the pentamer and monomer instruct IgM hinge engineering for therapeutic and diagnostic applications^50–52^.

## Methods

### Protein expression and purification

Full-length IgM pentamer. Human IgM pentamer from myeloma (Jackson ImmunoResearch, RRID: AB_2337048) was purified using a Superose 6 10/300 GL column to remove trace amounts of dimers and hexamers. The protein was concentrated at 1.0 mg/ml in PBS.

IgM-Fc monomer and IgG1-Fc. Recombinant human IgM-Fc and IgG1-Fc were based on the extracellular parts of the membrane forms of the immunoglobulins. The IgM Fc monomer contained Cμ2-Cμ4 domains followed by the stalk normally linking the extracellular domains to the transmembrane region (residues 228-569) and included a mutation of C444S to prevent oligomerization and incorporation of fluorescent dyes at this position. IgG1 Fc contained Cγ2-Cγ3 followed by the stalk (residues 227-464) and included mutation of the hinge cysteine (C229S), which also interfered with labelling. None of the mutations affected the expression, dimerization, or stability of the IgM and IgG1 proteins.

To introduce fluorescent labels at sites whose distances could distinguish the mode of Cμ4 orientation and dynamics, residues E526 (in the EF helix) and T556 (at the end of the G strand) at the side and bottom of Cμ4 of IgM, respectively, were mutated to cysteines. Correspondingly, amino acids S415 and S444 in Cγ3 of IgG1 were mutated to cysteines. To disrupt Cμ4 dimerization, histidine at position 518 in the Cμ4 domain was mutated to glutamic acid. The Cμ4 dimerization was stabilised by adding a C-terminal self-dimerising coiled-coil (EIAQLEYEISQLEQEIQALES)^53^. These two latter changes were introduced into IgM E526C. All constructs were cloned into pHLSec vector, in a frame with an N-terminal signal peptide sequence for secretion and with a C-terminal twin Strep Tag for purification of the secreted proteins.

The proteins were expressed by transient transfection of the plasmids into adherent 293 T cells using PEI (1.0 μg/ul, 1:4 (w/w) DNA to PEI). Transfected cells were cultured for 5 days and the media containing the proteins were collected, filtered and applied to a Strep-Tactin sepharose column (IBA Life Sciences). Bound proteins were eluted with desthiobiotin and purified using FPLC.

### Cryo-EM sample preparation and imaging

Full-length IgM pentamer. Purified IgM pentamer samples were diluted to 0.25 mg/ml and 4 μls were applied to amylamine glow-discharged holey carbon copper grids (Quantifoil, R2/2) in the environmental chamber of a Vitrobot Mark IV (FEI/Thermo) at 4 °C and 100% humidity, and the grid was blotted with filter paper and then plunge frozen in liquid ethane at liquid nitrogen temperature. The IgM cryo grids were screened on Talos Arctica microscope (FEI/Thermo) at 200 kV and data collection performed on a Titan Krios microscope (FEI/Thermo) at 300 kV using EPU software. Movies were recorded on a Falcon 3 camera in counting mode with a total dose of 34.2 electrons per Å^2^ fractionated over 30 frames (dose rate 0.51 e^−^/Å^2^/s) with a 1.09 Å pixel size and a defocus range between −1 to −5 μm.

IgM Fc monomer. Purified IgM Fc monomer (with coiled-coil extension at C-terminus) were diluted to 0.2 mg/ml and cryo-EM grids were made and screened using the same approach as the IgM pentamer above. Data were collected on a Titan Krios microscope (FEI/Thermo) at 300 kV equipped with a Gatan Imaging Filter (GIF) using EPU software. The slit width of the energy filter was set to 20 eV. Movies were recorded on a K2 camera in counting mode with a total dose of 66 electrons per Å^2^ fractionated over 40 frames (dose rate 8.25 e^−^/Å^2^/s) with a 0.839 Å pixel size and a defocus range between −1 to −4 μm.

### Cryo-EM data processing and model docking

Full-length IgM pentamer. The workflow of data processing is shown in Supplementary Fig. 1. A total of 43,530 movies were collected and imported into Scipion (v 2.0) in streaming mode. Raw movies were then aligned and dose-weighted by Motioncor2^54^, followed by CTF estimation by CTFfind v 4.1.13 on the dose-weighted micrographs^55^. 1500 particles were manually picked in 50 micrographs acquired at various defocus values for model training by crYOLO^56^, which was then used for picking particles from all the micrographs. A total of 1,819,931 picked particles were extracted and exported by Relion particle extraction and export, with a box size of 600 pixels. The following image processing steps were conducted in CryoSPARC (v 3.2.0)^57^. All raw particles were imported into CryoSPARC and after two rounds of 2D classification, 546,583 particles were selected for further analysis. Five ab initio classes were calculated and used as initial models for heterogeneous refinement. 301,286 particles in the two best classes were then combined and subjected to non-uniform refinement^58^. This resulted in a map with a 4.32 Å global resolution calculated by Phenix^59^. Sharpening with B factor of −197 Å^2^ was applied to boost the high-resolution features of the map.

3D variability analysis (3DVA) was conducted to explore the flexibility within the IgM molecule (Supplementary Fig. 4)^30^. The 301,286 particles contributing to the final refined map were downsampled by a factor of 2 (box size = 300 pixels), and three components were calculated. The volume series shown in the Supplementary Movie 1 and Supplementary Movie 2 is the first variability component. At the same time, the particles were clustered into six subsets by 3DVA, five of which were individually reconstructed by non-uniform refinement to improve particle alignments and resolution. Particle subtraction and focused refinement for each class were then calculated to improve the alignment and resolution locally at subunit 1, especially the F(ab’)_2_ region.

Models (pdb id 6KXS, 4JVU, 2AGJ) were rigidly docked and combined into the maps and geometry minimisation was performed to regularise the models, followed by rigid body refinement using res-space refinement in Phenix^59^. The maps and models were displayed in Chimera v 1.13.1^60^. Details on data recording and processing of the FL-IgM pentamer are summarised in Supplementary Table 2.

IgM Fc monomer. 14,815 movies were collected, which were aligned and dose-weighted by Motioncor2 in Relion3.1^61^, followed by CTF estimation by CTFfind v 4.1.13. 7,411,324 particles were picked from 13,012 selected micrographs by a trained model in crYOLO and extracted with a box size of 50 pixels (pixel size=3.356 Å). The particle stack was cleaned by 2D classification in CryoSPARC for two rounds, with 961,072 particles selected and re-extracted with a box size of 256 pixels (no binning, pixel size=0.839 Å), which were then processed with Relion for 3D initial model, 3D auto-refine, Bayesian polishing and CTF refinement^61,62^. The polished and CTF-refined particles were transferred back to CryoSPARC for non-uniform refinement with C2 symmetry. The final map has a global resolution of 3.6 Å and a B factor of 185 Å^2^.

Initial model of mIgM-Fc is obtained from chain E and F from the IgM pentamer cryoEM structure (pdb id 6KXS). Tailpieces were removed and single mutation C414S was made on both chains. Real-space refinement of the model against the mIgM-Fc cryo-EM map was then conducted in Phenix (v1.19.2) and manual refinement in Coot (v0.9.6). map-model FSC at 0.5 cut-off is 3.79 Å. The maps and models were displayed in Chimera v 1.13.1^60^. Details on data recording and processing of IgM Fc monomer are summarised in Supplementary Table 3.

### Quantification of the range of Fab motion in subunit 2-5

The whole IgM map is composed of a series of cross-sections along any orientation, as shown in Supplementary Fig. 8a. The cross-sections are opened in ImageJ (v 2.1.0), converted to 8-bit, and filtered with an isotropic 3D Gaussian function with ${{{{{\rm{\sigma }}}}}}=3$ px to reduce the high-frequency noise which would influence the accuracy for the following measurements.

Supplementary Fig. 8b–e are the middle cross-sections of all the F(ab’)_2_ clouds along both in-plane and out-of-plane directions, and thus represent the maximum angle that the Fab can reach in each subunit. The curvatures of the inner and outer arc indicate that they share the same centre, which is the hinge region of F(ab’)_2_ motion at the C-terminus of the Cμ2 domain. This is confirmed by fitting the centre of each arc into a circle (dashed circles in Supplementary Fig. 8b–e), and the blue spots shown in the cross-sections are the shared centre of the arcs at Cμ2/Cμ3 interface.

The Fab motion range can be quantified based on the grey values on the inner (Fab constant region) and outer (Fab variable region) arcs on the cross-sections. One can see from the cross-sections that for either the inner or outer arc the grey value at the centre is the highest (brighter), and it becomes darker with the length of the arc extending both ways and finally falls to the level of the background. Supplementary Fig. 9a and Supplementary Fig. 9b demonstrate the approach to measure the grey values of the arcs in a series of in-plane angle ${{{{{\rm{\alpha }}}}}}$ and out-of-plane angle ${{{{{\rm{\beta }}}}}}$ for subunit 4. The line profiles are plotted using the ‘plot profile’ function in ImageJ^63^. Three peaks are present in the plots, corresponding to the densities of Cμ2, Fab constant domain (inner arc) and Fab variable domain (outer arc). The values of all three peaks decrease monotonically with ${{{{{\rm{\alpha }}}}}}$ or ${{{{{\rm{\beta }}}}}}$ increases, and at some point, the peaks of inner and outer arc are not significantly higher than the background (black curves).

The criteria used in this study for the peak grey value to be considered as above noise is described in Eq. (1)

1 $$\frac{{{{{{\rm{grey\; value}}}}}}-\overline{{{{{{\rm{BKG}}}}}}}}{{{{{{{\rm{\sigma }}}}}}}_{{{{{{\rm{BKG}}}}}}}} \, \ge \, 2$$

where $\overline{{{{{{\rm{BKG}}}}}}}$ is the mean of the background noise, and ${{{{{{\rm{\sigma }}}}}}}_{{{{{{\rm{BKG}}}}}}}$ is the standard deviation of the background noise. The ${{{{{\rm{\alpha }}}}}}$ or ${{{{{\rm{\beta }}}}}}$ at which the result of the above equation equals 2 is regarded as the boundary of the Fab moving range. Supplementary Fig. 9c to Supplementary Fig. 9f summarises the results for subunit 2-5 along both positive and negative directions. Six data points were collected on six consecutive cross-sections around the middle section for each subunit. These results quantitatively demonstrate that the in-plane flexibilities of Fab is in the range of $50^\circ -65^\circ$ and $70^\circ -80^\circ$ along the out-of-plane direction, and there is no significant difference among subunit 2-5, or along positive or negative directions.

### Labelling of IgM monomers and IgG1 Fc proteins and smFRET microscopy

The proteins were labelled at the introduced cysteines with FRET pair dyes using Alexa Fluor 555 C2 maleimide and Alexa Fluor 647 C2 maleimide (both from ThermoFisher). Before labelling, proteins were buffer-exchanged into 50 mM Tris (pH 6.8), 140 mM NaCl and 20 μM TCEP. A five times molar excess of each dye, prepared in labelling grade DMF (Lumiprobe), was simultaneously added to the protein and the mixture was incubated with shaking at room temperature for 2 h. Labelled proteins were purified by size exclusion chromatography using a Superdex 200 10/300 GL column. Dye incorporation was examined by SDS-PAGE gels using ImageQuant imaging system (Amersham) and by absorbance measurements. Calculation of dye incorporation generally showed a near 1:1 incorporation of each dye with more than 90% protein labelled. No significant labelling was observed of control proteins lacking the introduced cysteines.

Borosilicate glass coverslips (VWR) were treated with acid piranha solution (a 3:1 mixture of concentrated sulphuric acid with hydrogen peroxide) in glass jars for 15 min. The coverslips were extensively washed with distilled water followed by 2 washes with ethanol and one final wash again with distilled water. Coverslips were then washed three times with acetone to remove all traces of water. The coverslips were then incubated with 2% (v/v) silane (3-aminopropyltriethoxysilane, Sigma) in acetone. After 2 min, coverslips were rinsed thoroughly with distilled water. The coverslips were dried under argon and mounted on microscope slides using two 0.12 mm-thick strips of double-sided adhesive tape (Sigma). The two strips were separated by a gap of about 4 mm creating a channel for the application of the samples.

In order to specifically attach the labelled proteins, the slide-mounted coverslips were first coated with biotin-PEG as follows: 2 mg biotin-PEG SVA and 150 mg of mPEG-SPA (both from Laysan Bio) were dissolved in 1 ml of 0.1 M NaHCO3 (pH 8.2) and applied to the channels between each coverslip and the slide. The coverslips were left with the coverslip side down in a humidified chamber for 3 h. Afterwards, the channels were washed extensively with distilled water and dried under argon. The coverslips were either immediately used or stored under vacuum in a desiccator for up to 3 weeks. Prior to the experiments, the channels were coated with streptavidin (1.0 mg/ml in PBS) for 30 min. After washing out unbound streptavidin, the channels were loaded with biotinylated anti-IgM or anti-IgG antibodies serving as anchors for the fluorescent proteins. IgM Fc was captured using monoclonal antibodies directed against the Cμ2 domain of IgM (anti-human IgM MU53 and HB57, generous gifts from Patricia Mongini^64^, Feinstein Institutes for Medical Research). IgG1 Fc proteins were captured using anti-IgG1 antibodies directed against the Cγ2 domain (Bio-Rad, anti-human IgG clone MK1A6).

Labelled proteins were applied to channels containing the antibodies at an appropriate dilution to achieve a sparse surface density suitable to imaging of individual fluorescent spots. After washing, PCA/PCD/Trolox oxygen scavenging buffer (0.385 mg/ml Protocatechuic acid, 7 μg/ml Protocatechuate 3,4-dioxygenase, 0.3 mg/ml Trolox (all from Sigma) in Tris-buffer saline pH 8) was introduced for single molecule imaging.

The samples were imaged on an Olympus IX-81 TIRF microscope with iLAS laser illumination system, 100x objective, image splitter and Andor iXon camera (CAIRN imaging). The sample was initially imaged by acquiring a donor image (illumination at 552 nm and emission at 565–615 nm, a FRET image (illumination at 552 nm and emission at >650 nm) and a directly excited acceptor image (illumination at 637 nm and emission at >650 nm), followed by time-lapse imaging at 30 ms per frame using 552 nm excitation and simultaneous imaging of the donor and acceptor (FRET) emission.

### FRET data analysis and dye distance calculation

Single-molecule FRET was analysed similarly as described previously^65,66^ using automated image processing in Matlab (Mathworks) and semi-automatic identification of single-molecule fluorescent traces. All imaging channels were spatially aligned using multispectral beads. Background in each channel was subtracted using background images generated by medium filtering at each pixel position across many experimental images containing sparse single-molecule fluorescence spots. Spots containing both donor and acceptor molecules were identified using initial snapshots of the acceptor fluorescence and recovered donor fluorescence, which was calculated as Eq. (2)

2 $${{{{{\rm{Recovered}}}}}}\; {{{{{\rm{donor}}}}}}\; {{{{{\rm{fluorescence}}}}}}={{{{{\rm{D}}}}}}+{{{{{\rm{F}}}}}}/{{{{{\rm{\gamma }}}}}}$$

where D is the donor image intensity (donor excitation and donor emission), F is the FRET image intensity (donor excitation and acceptor emission) and γ is the factor that relates fluorescence intensity of F to D, measured from donor recovery after acceptor photobleaching of molecules exhibiting non-zero FRET in each experiment as Eq. (3)

3 $${{{{{\rm{\gamma }}}}}}=({{{{{{\rm{F}}}}}}}_{{{{{{\rm{before}}}}}}}-{{{{{{\rm{F}}}}}}}_{{{{{{\rm{after}}}}}}})/({{{{{{\rm{D}}}}}}}_{{{{{{\rm{after}}}}}}}-{{{{{{\rm{D}}}}}}}_{{{{{{\rm{before}}}}}}})$$

FRET efficiency of individual molecules over time from time-lapse images was calculated as Eq. (4)

4 $${{{{{\rm{E}}}}}}=({{{{{\rm{F}}}}}}-{{{{{\rm{\alpha }}}}}}{{{{{\rm{D}}}}}}-{{{{{\rm{\beta }}}}}}{{{{{\rm{A}}}}}})/({{{{{\rm{F}}}}}}-{{{{{\rm{\alpha }}}}}}{{{{{\rm{D}}}}}}-{{{{{\rm{\beta }}}}}}{{{{{\rm{A}}}}}}+{{{{{\rm{\gamma }}}}}}{{{{{\rm{D}}}}}})$$

where D, F and A are the mean pixel intensities of a 5 × 5 pixel area of the fluorescent spot in the donor, FRET and directly-excited acceptor channels, respectively, α is the fraction of donor fluorescence bleeding into the FRET channel (α = F/D in donor-only molecules), β is the fraction of FRET channel fluorescence attributable to direct 552 nm excitation of the acceptor (β = F/A in acceptor-only molecules), and γ is as explained above. The correction factors α, β and γ were determined in each experiment as the mean of these factors from at least five images using the appropriate controls. Typically, α = 0.09, β = 0.08, and γ = 1.8. Only molecules that showed single-step donor or acceptor photobleaching were included in the analysis and were curated manually. Mean FRET was calculated by the fitting of one or two gaussians into the histogram of the FRET values aggregated from all time points of all the molecules analysed.

To compare the measured FRET values to FRET estimated from the distances of the dyes attached to the proteins at the indicated positions, we calculated the mean dye accessible volume for each labelled position as described^67^. Briefly, models of the crystal structure of the Cμ4 (pdb id: 4JVW), crystal structure of the IgG1 (pdb id: 3DO3) and Cryo-EM structure of the IgM pentamer (pdb id 6KXS) were used to virtually attach the donor and acceptor dyes to the Cβ atoms of the appropriate residues after the original sidechains had been removed. The accessible dye volume clouds were generated using the FPS software using the settings stated in Supplementary Table 4^67^.

The accessible volume was then used to derive the mean inter-dye distance (FRET-weighted) and the predicted FRET value for each labelling situation, with R_0_ for the Alexa Fluor 555 – Alexa Fluor 647 pair set to 5.1 nm.

### Reporting summary

Further information on research design is available in the Nature Research Reporting Summary linked to this article.

## Supplementary information

Supplementary Information

Description of Additional Supplementary Files

Supplementary Movie 1

Supplementary Movie 2

Reporting Summary

## Data Availability

The structural data that support the findings of this study have been deposited in the Protein Data Bank and EM Data Bank. The pentameric IgM EM map displayed in Fig. 1 has entry number EMD-13921. The five IgM conformations shown in Fig. 3 are conformation 1 (EMD-15375, pdb 8ADY), conformation 2 (EMD-15376, pdb 8ADZ), conformation 3 (EMD-15377, pdb 8AE0), conformation 4 (EMD-15380, pdb 8AE3), conformation 5 (EMD-15379, pdb 8AE2). The monomer IgM-Fc map and model have entry numbers 7QDO and EMD-13922. 43,332 aligned and dose-weighted micrographs haven been deposited on EMPIAR (id EMPIAR-11077). Sample data of single-molecule FRET have been provided for data analysis at Github (https://github.com/ptolar/single-molecule-FRET-trace-analysis). Source data are provided with this paper. All the models used in this study (pdb id 6KXS, 4JVU and 2AGJ) are available in PDB and cited in the paper. Source data are provided with this paper.

## Source data

Source Data

## Code availability

Custom MATLAB code to analyse single-molecule FRET from time-lapse images is available at Github (https://github.com/ptolar/single-molecule-FRET-trace-analysis).
