# Supplementary material for: Cryomicroscopy reveals the structural basis for a flexible hinge motion in the immunoglobulin M pentamer
Source: Nat Commun. 2022 Oct 23;13:6314. doi: 10.1038/s41467-022-34090-2 (PMC9588798; doi:10.1038/s41467-022-34090-2)
Supplement: Supplementary file 1 — Supplementary Information [file 41467_2022_34090_MOESM1_ESM.pdf]

Supplementary Information

Cryomicroscopy reveals the structural basis for a flexible hinge motion in the Immunoglobulin M Pentamer

Qu Chen[1], Rajesh Menon[2], Lesley J. Calder[3], Pavel Tolar[2,4*] and Peter B. Rosenthal[3*]

[1]Structural Biology Science Technology Platform, The Francis Crick Institute, 1 Midland Road, London, NW1 1AT, UK.

[2]Immune Receptor Activation Laboratory, The Francis Crick Institute, 1 Midland Road, London, NW1 1AT, UK.

[3]Structural Biology of Cells and Viruses Laboratory, The Francis Crick Institute, 1 Midland Road, London, NW1 1AT, UK.

[4]Institute of Immunity and Transplantation, University College London, Rowland Hill Street, London, NW3 2PP, UK.

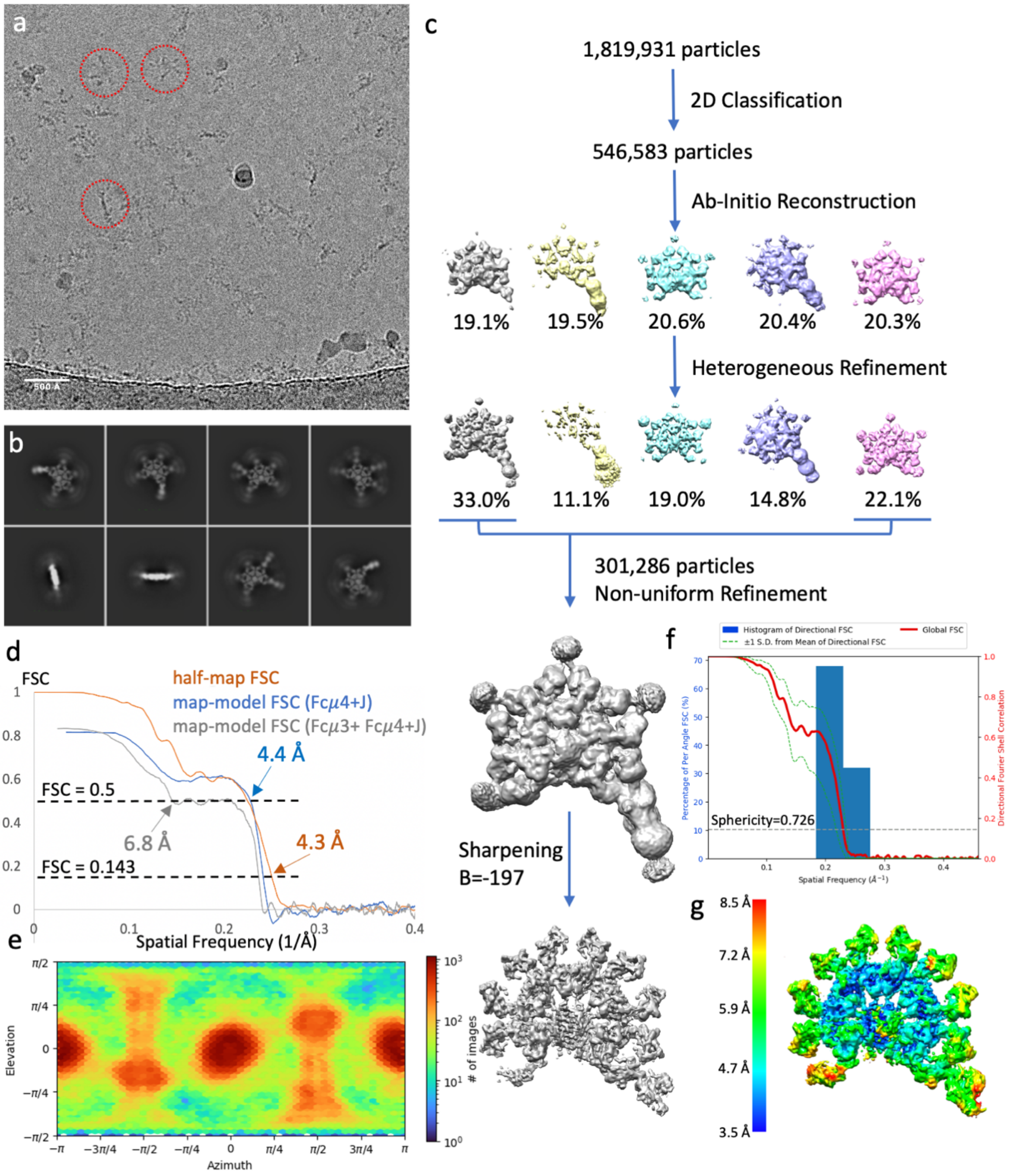

Supplementary Fig. 1. Workflow of single particle analysis of FL-IgM. (a) A typical micrograph with front and side views of IgM highlighted in red dotted circles. 43,530 micrographs in total. (b) Representative 2D classes. (c) Flow chart for data processing. (d) Gold standard Fourier shell correlation (FSC) and map-model FSC curves with estimated resolutions calculated in Phenix. The model used for calculations of map-model FSC is pdb id 6KXS. (e) Angular distribution of the particles used in the final homogeneous refinement. (0,0) is the front view of IgM molecule, which is the most populated orientation. Side views are also present with slightly less frequency. (f) 3DFSC histogram of the refined map. (g) Sharpened map showing local resolution estimation of the higher-resolution core region. Local resolution calculations performed in CryoSPARC.

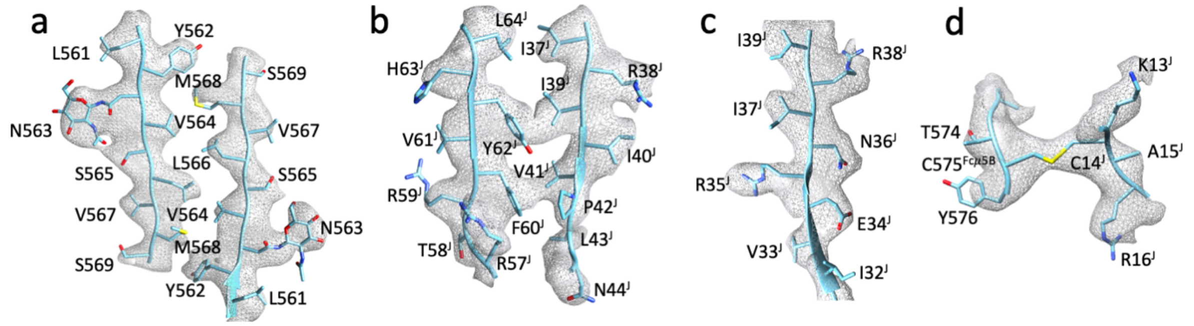

Supplementary Fig. 2. Density maps of core regions in the structure. (a) Densities of two β strands in the tailpiece assembly. (b) Densities of the two β strands at the centre of J chain. (c) Densities of a segment of the haipin-1 loop of J chain. (d) Density of a disulfide bond between Fcμ chains and the J chain.

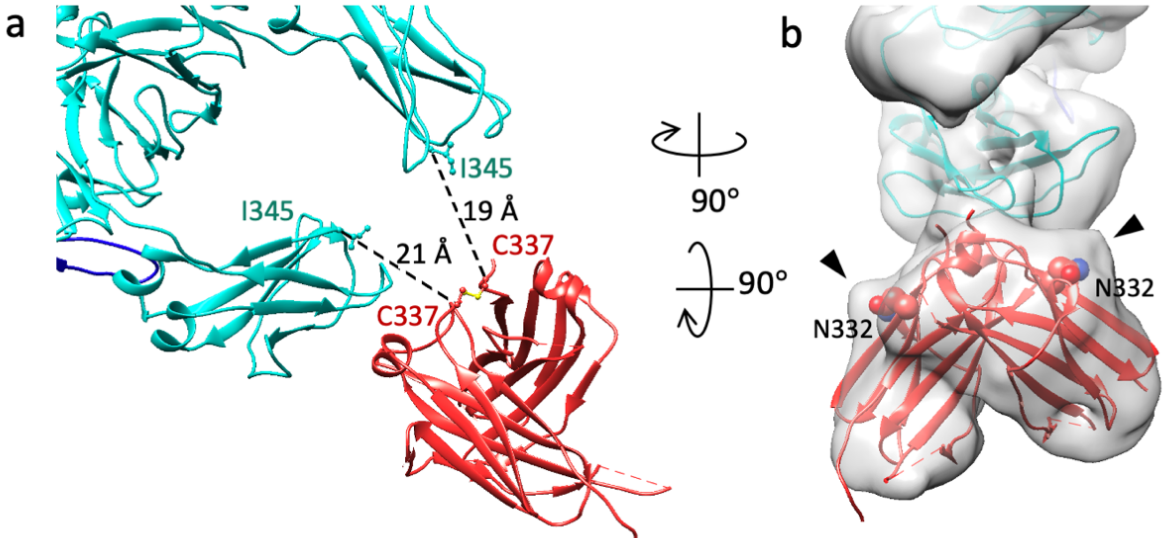

Supplementary Fig. 3. Location of Cμ2 domain at IgM subunit 1. (a) Distances between Cμ3 and Cμ2 domain in subunit 1 measured from the a-carbon of ILE 345 on Cμ3 to the a-carbon on Cysteine 337 on Cμ2 (atoms are highlighted by ball and sticks). (b) The map density for Cμ2 at threshold 0.42 (unsharpened map). Arrows (also see Supplementary Fig. 5) identify protrusions on each subunit corresponding the N-linked glycosylation at N332 (atoms in N332 side-chain are highlighted by spheres, carbohydrate residues not shown).

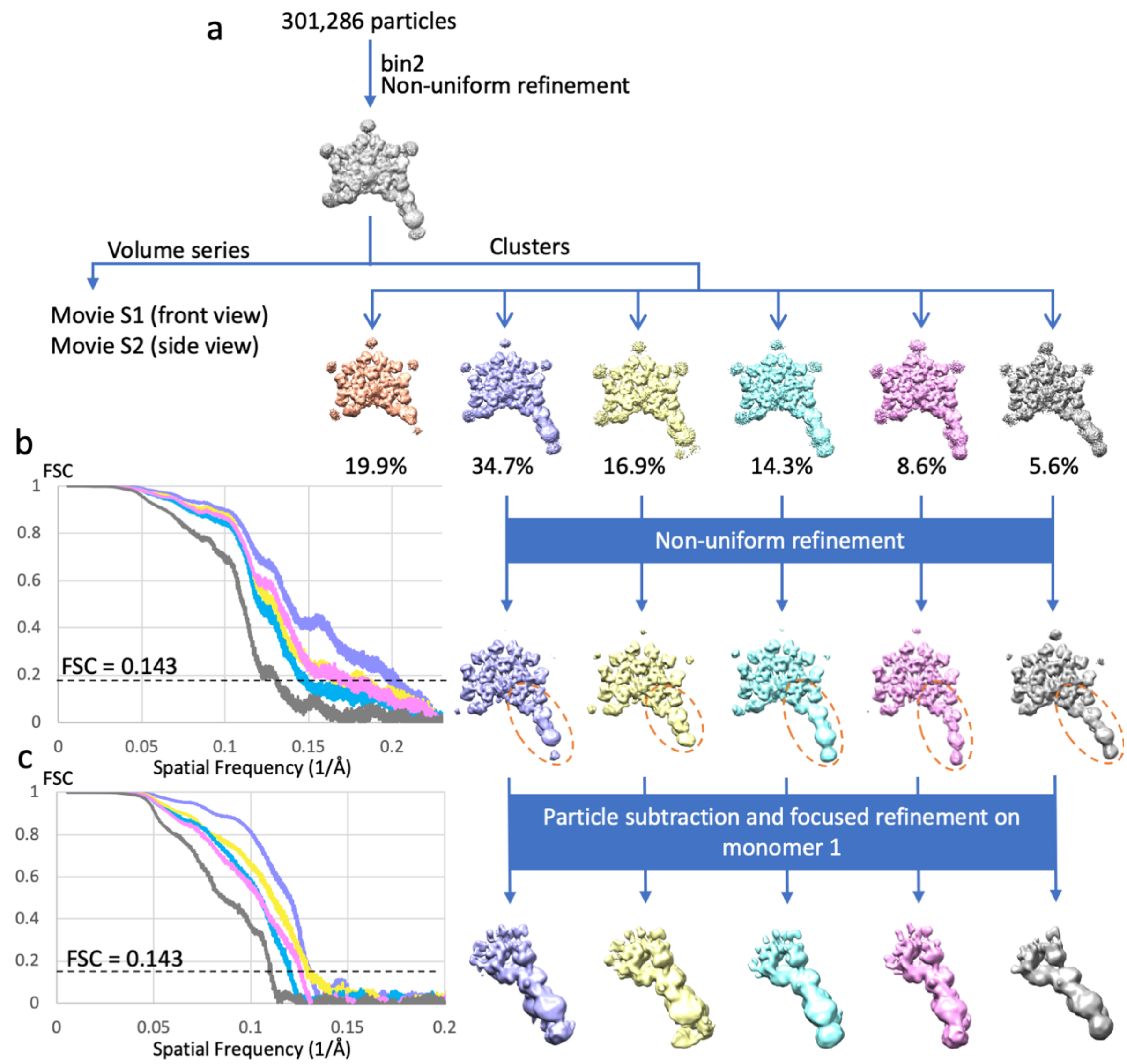

Supplementary Fig. 4. Workflow of 3D variability analysis (3DVA) and focused refinement on subunit 1. (a) The particle dataset was binned with factor of 2 and refined by non-uniform refinement (CryoSPARC). Six subsets were classified by the 'cluster' function in 3DVA and five of them show characteristic features at $F(ab')_2$ in subunit 1. Each cluster was subjected to non-uniform refinement. (b) FSC curves for the five subclasses, coloured as for the maps. The global resolutions of the subclasses based on FSC are in the range from 5-9 Å. Masks for calculations include Cμ4-Cμ2 for all five subunits and Fabs of subunit 1. (c) FSC curves for the five local-refined maps. Cμ4 to Fabs of subunit 1 are included in the masks for the calculations. The local refined maps are globally 8-9 Å resolution.

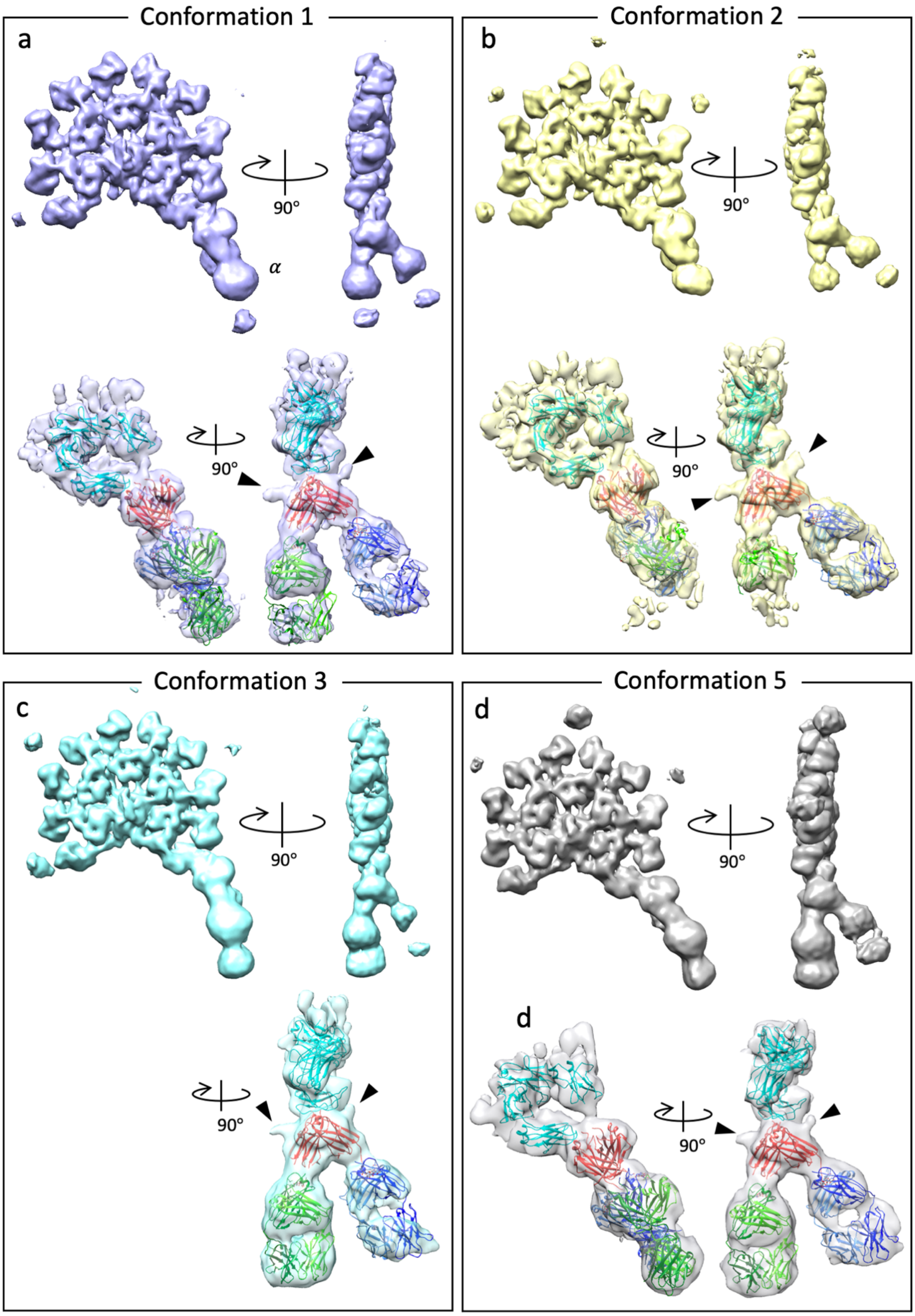

Supplementary Fig. 5. Conformations of F(ab')$_2$ at IgM position 1. (a-d) Conformation 1-3 and 5 classified from 3DVA and corresponding focused refinement (conformation 4 is in Fig. 3a-c). pdb id of

docked models are 6KXS (cyan, subunit 1 only), 4JVU (red, Cμ2) and two 2AGJ (green and blue, Fab). The heavy chains are dark green/blue and light chains are light green/blue. The two black arrowheads in each conformation indicate the density of oligosaccharide (not shown in model) at N332. Except for the Fab variable domain in conformation 2, Fab models can be rigidly docked into all the Fab/Fab' densities based on the two subunits of the constant domain (Cμ1 and CL). The map-model $FSC_{0.5}$ of all five focused refined maps are conformation 1 (a), 20.0 Å; conformation 2 (b), 19.6 Å; conformation 3 (c), 20.2 Å; conformation 4 (Fig. 3b), 19.4 Å and conformation 5 (d), 20.0 Å. Threshold values of the maps are 2, 2.1, 2.1 and 2.2 for global refined maps (top map in each panel) and 1.9, 2.1, 1.9 and 1.75 for focused refined maps (lower map in each panel). B factors used for sharpening of all focused-refined maps are -500 $Å^2$. The assigned orientation, where heavy chains are located outside and light chain inside, has higher cross-correlation scores than the other way around.

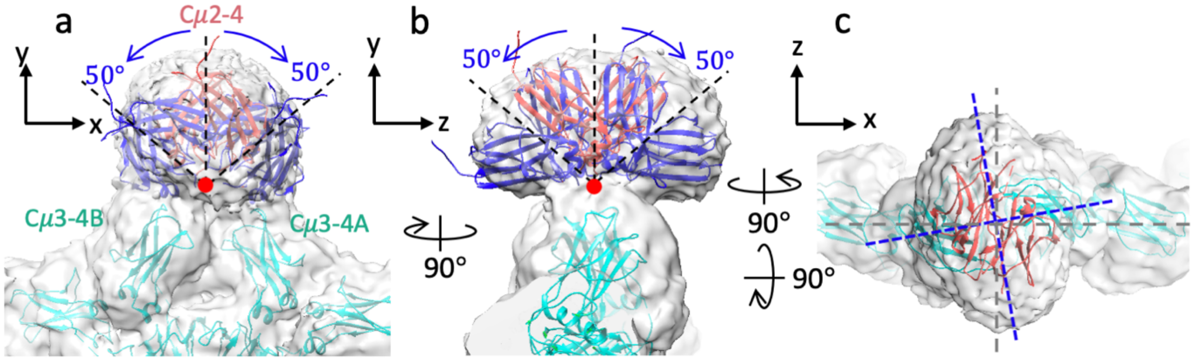

Supplementary Fig. 6. Cμ2 densities at subunit 2-4. (a-b) Front and side view Cμ2 density of subunit 4 at threshold 0.1. Three Cμ2 crystal models 4JVU are docked in (a) – the central one (red) and clockwise and anticlockwise tilted at 50° at the pivot point (red spot) along in-plane direction. The models docked in (b) are 0 tilt (red), and clockwise and anticlockwise tilted at 50° at the pivot point (red spot) along the out-of-plane direction. The distance between C337 (Cμ2) and I345 (Cμ3) for the five different Cμ2 positions are all within the range of 18-24 Å, shorter than the extended length of eight residues (3.5 Å per residue in average). (c) Top view of Cμ2 density of subunit 4. The two symmetry axes (blue dashed axes) are about 10° tilted from the actual in-plane and out-of-plane direction (grey dashed axes).

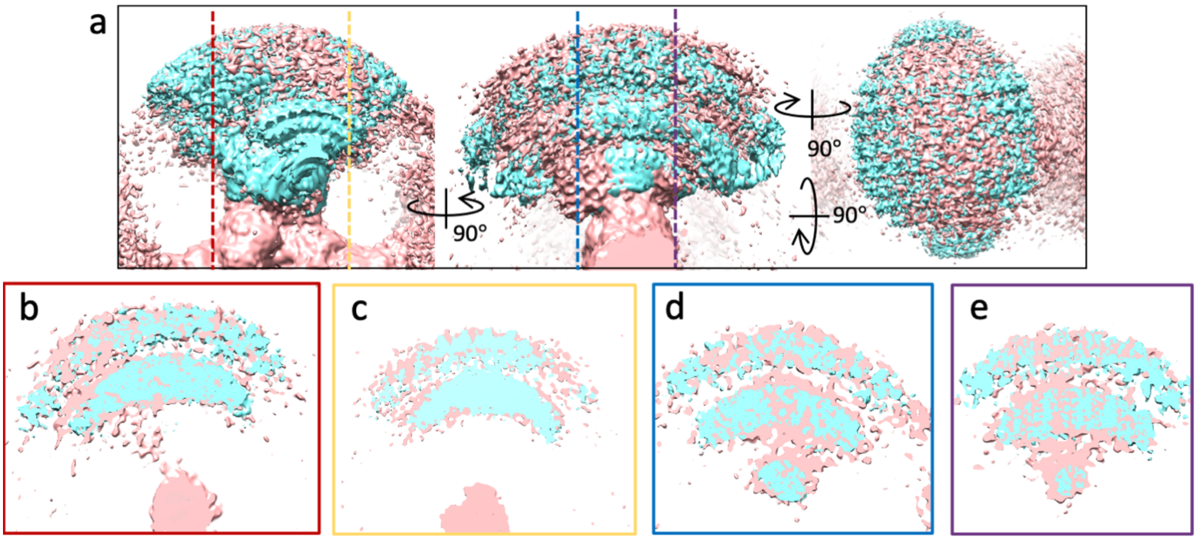

Supplementary Fig. 7. Additional cross-sections for the simulated and experimental densities. (a) Overlay of the experimental (pink, threshold 0.025) and simulated map (cyan) of F(ab')$_2$, same as Fig. 4c-e (lower panels). (b-e) Cross-sections of the overlayed maps indicated by the dotted lines in (a). The simulated maps are generated by using the command 'molmap' at 5 Å resolution in the program Chimera with all the models shown in Fig. 4c-e summed.

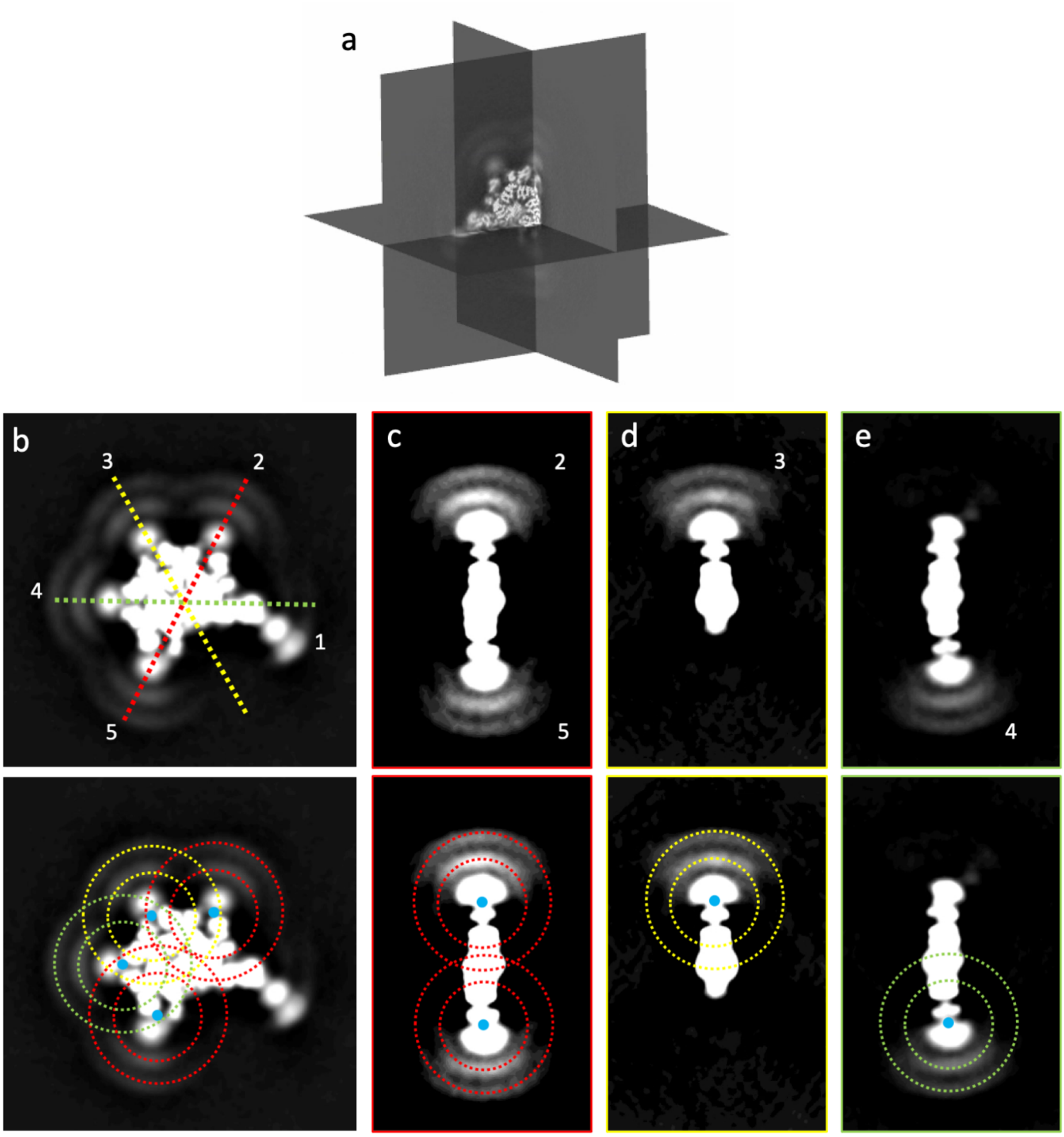

Supplementary Fig. 8. Identifying the rotational centre for F(ab')$_2$ pivot motion in the cross-section views. (a) The whole IgM map is composed of a series of cross-sections along any orientation. Three orthogonal cross-sections are shown in (a). (b) The cross-sections which run through the central plane of the IgM Cμ4-Cμ3 platform. Subunit 1-5 are labelled on the top panel and the double-layer clouds in subunit 2-5 are clearly visible. In the bottom panel, the dotted circles are running through the centre of the inner and outer arc for each subunit and the shared centre for each pair of arcs are identified by the blue dots, which is the rotational centre (hinge). (c-e) The cross-sections through the dotted lines in (b, top) with the same colouring, showing the out-of-plane motions. The rotational centres are identified by the same principle described in (b).

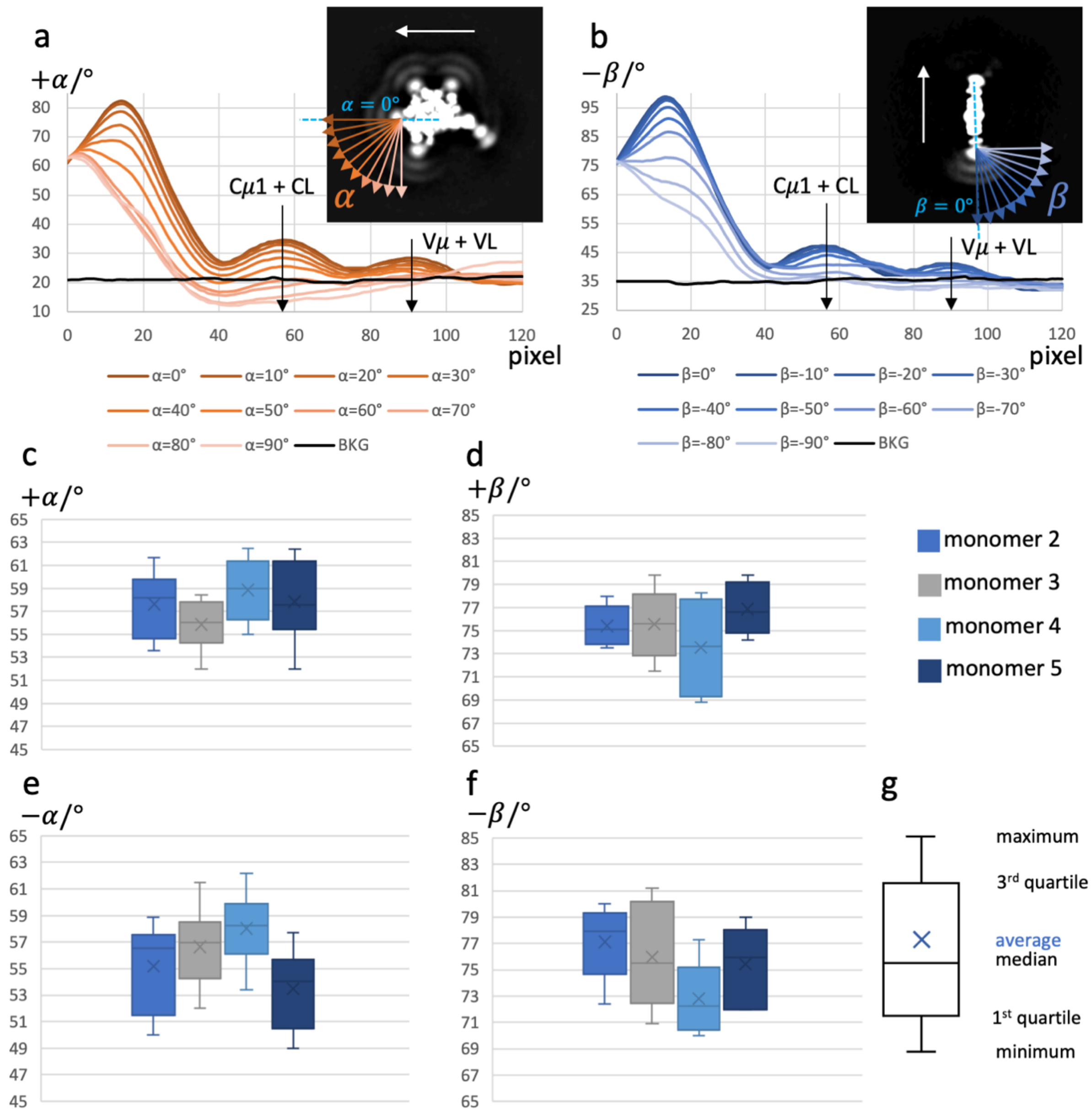

Supplementary Fig. 9. Quantification range of Fab motion in subunit 2-5. (a) A series of line profiles from $\alpha = 0°$ to 90° with 10° interval for subunit 4 on the front-view cross-section, as indicated by the arrows with the same colour codes. (b) A series of line profiles from $\beta = 0°$ to -90° with 10° interval for subunit 4 on the side-view cross-section with the same colour codes as the arrows. The definitions of $\alpha/\beta = 0°$/positive and negative axes follow the same rules described in the legend of Fig. 3. The profile of background is plotted at a random position outside the IgM volume (white arrows in the cross-sections). For both in-plane and out-of-plane profiles, the radii of the inner and outer arcs are almost a constant regardless of α or β values, shown by the two black arrows in (a and b). The radii of curvature for inner (Cμ1 + CL) and outer arc (Vμ + VL) are 57 pixels (62 Å) and 92 pixels (100 Å). (c-f) Box plots for the maximum α and β (both positive and negative) for subunit 2-5. n=6 voxel values were measured for each subunit at each direction in a single 3D map (EMD-13921). (g) Legend for the boxplots in (c-f). Source data are provided as a Source Data file.

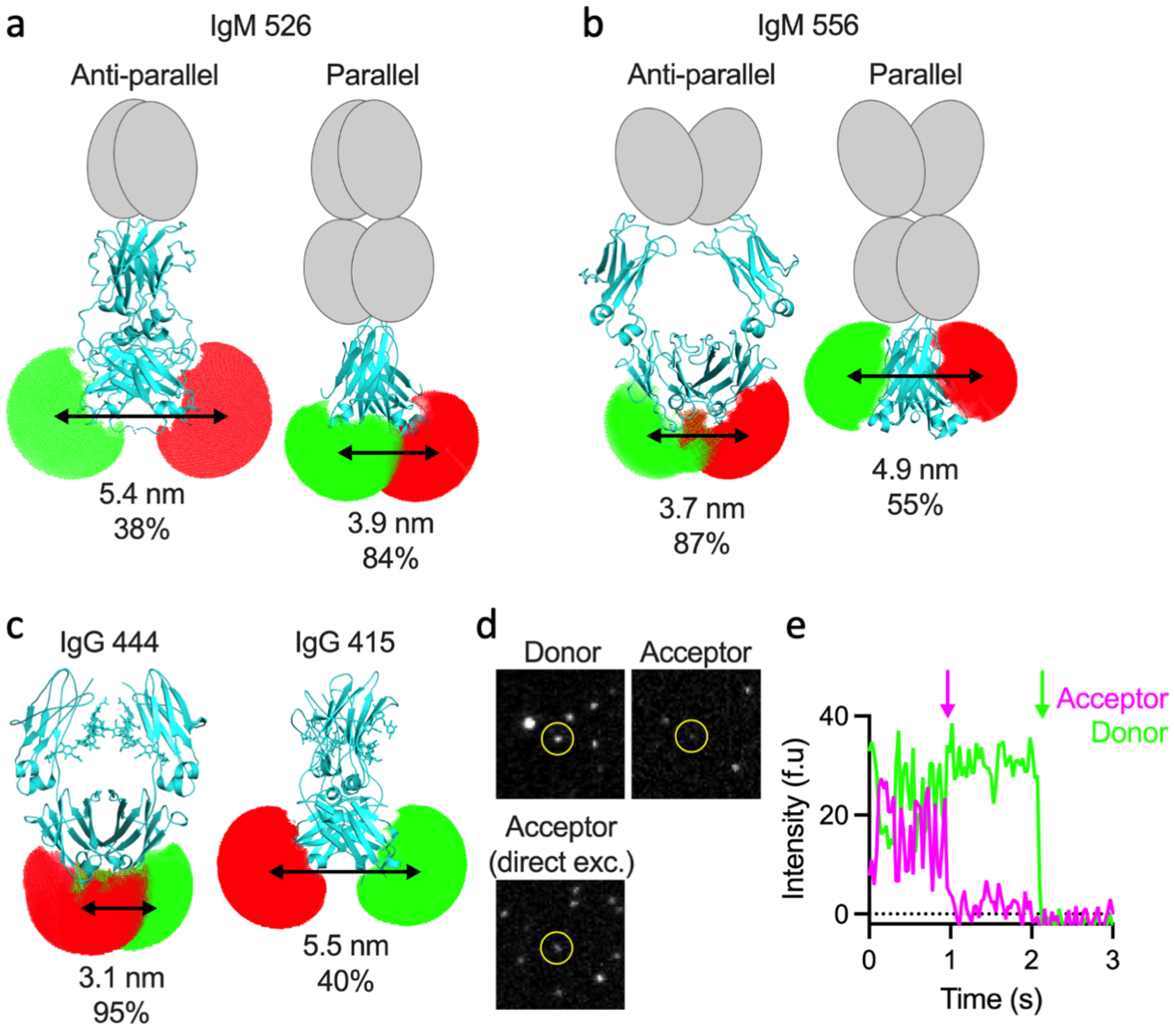

Supplementary Fig. 10. Constructs and imaging set up for single-molecule FRET studies of Cμ4 orientation and dynamics in IgM-Fc monomer. (a, b, c) Schematics of the IgM and IgG1 Fc constructs with modelled clouds of the donor (green) and acceptor (red) fluorophore positions when attached to the indicated sites on the proteins. The clouds were calculated using the illustrated structures of the IgM Fc from the IgM pentamer (the "anti-parallel" orientation, pdb id 6KXS), the isolated Cμ4 dimer (the "parallel" orientation, pdb id 4JVW), or the IgG1 Fc (pdb id 3DO3). Numbers below the structures show calculated mean donor-acceptor distances in nm and the predicted FRET efficiencies in percent. (d) An example of single-molecule FRET imaging. Images of the donor and acceptor channels acquired with donor illumination, and an acceptor image with direct acceptor illumination show the identification of single molecules containing one donor and one acceptor fluorophore. (e) Timelapse imaging of donor and acceptor channels simultaneously with donor illumination produces time-resolved fluorescent traces that are used to calculate FRET efficiency over time. Arrows indicate photobleaching of the acceptor (magenta) and the donor (green) fluorophore in single steps. Source data are provided as a Source Data file.

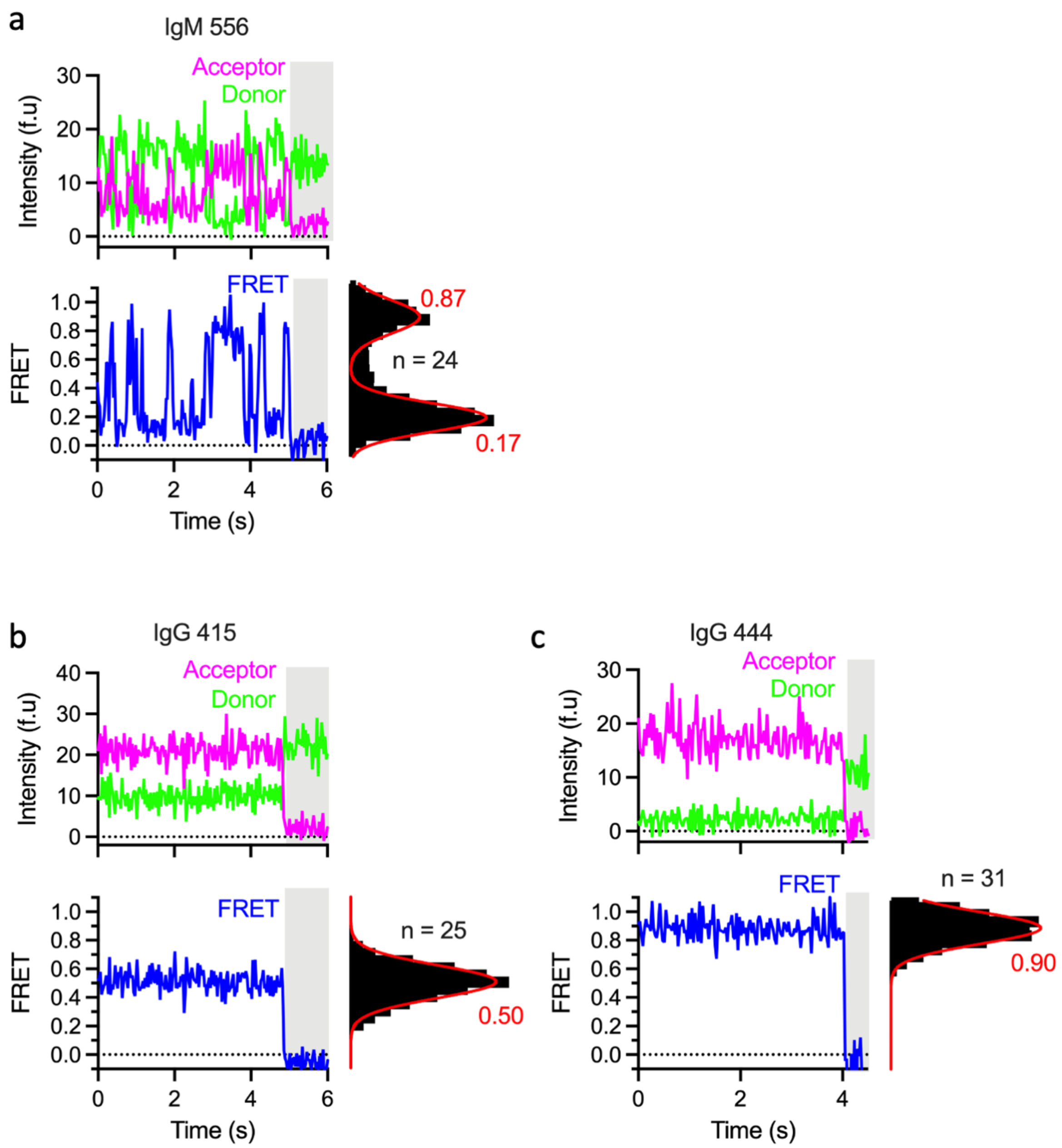

Supplementary Fig. 11. Single-molecule FRET resolves domain orientation and dynamics in IgM and IgG Fc monomers. (a) FRET donor (green) and acceptor (magenta) fluorescence intensity traces and the calculated FRET efficiency (blue) from a single molecule of a monomeric IgM-Fc construct labelled with the fluorophores at position 556 at the end of the G strand of Cμ4. The shaded area indicates when the acceptor molecule is photobleached. The histogram along the right FRET axis shows the distribution of the single-molecule FRET values for the n molecules measured. (b, c) FRET donor and acceptor intensity traces and calculated FRET efficiencies for IgG Fc constructs labelled on Cγ3 with the fluorophores at position 415 in the EF helix (b) and at position 444 at the end of the G strand (c). Source data are provided as a Source Data file.

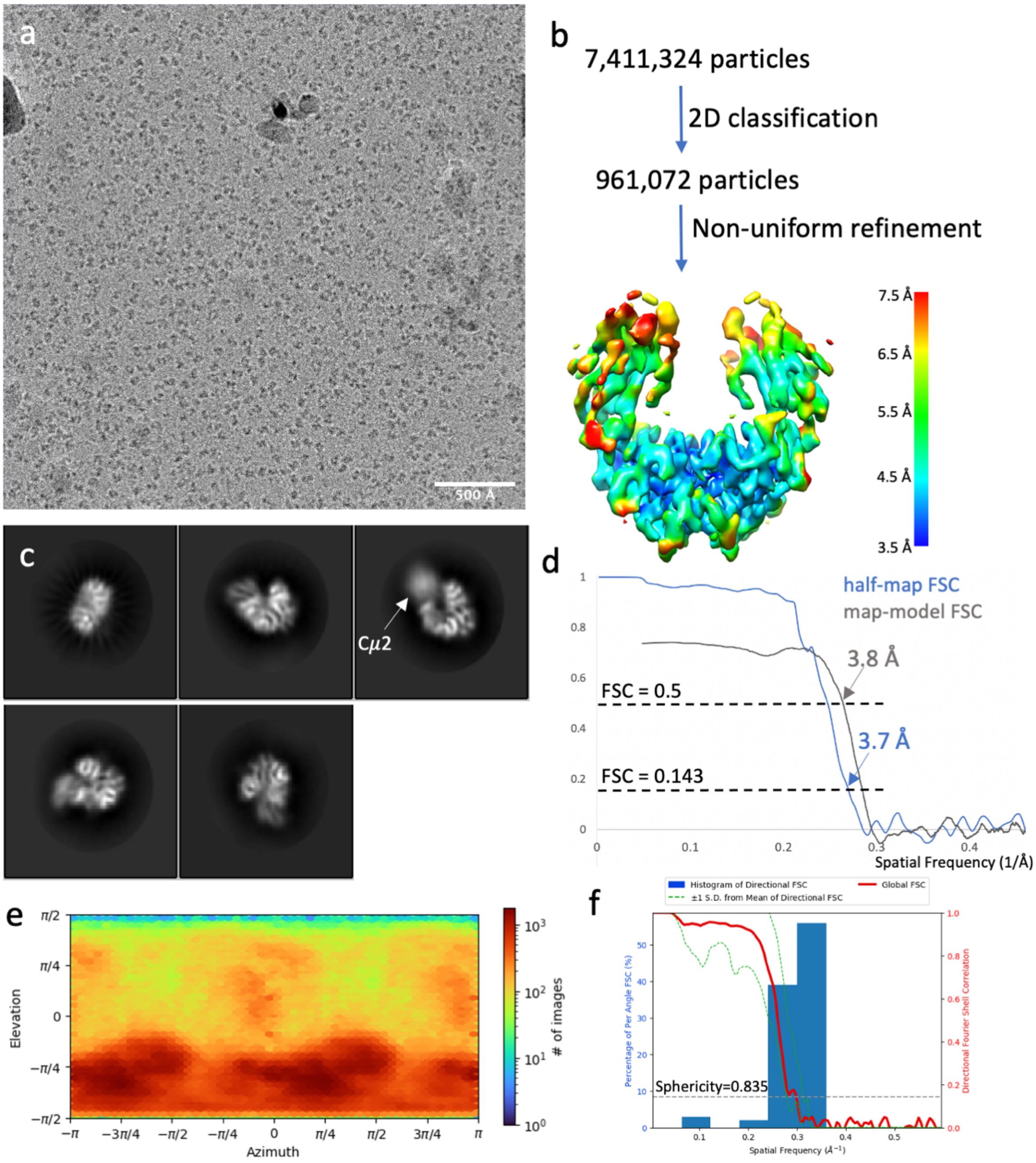

Supplementary Fig. 12. Workflow of single particle analysis of mIgM-Fc with C-terminal coiled-coil**.** (a) A typical field of view of the mIgM-Fc particles. 14,815 micrographs in total. (b) Flow chart for data processing from picked particles to final map. Non-uniform refinement is calculated with C2 symmetry. (c) Representative 2D classes, with Cμ2 domain indicated by a white arrow. (d) Gold standard Fourier shell correlation (FSC) and map-model FSC curves with estimated resolutions calculated in Phenix. Atomic model is shown in Fig. 5d. (e) Angular distribution of the particles contributed to the refined map. (f) 3DFSC histogram of mIgM-Fc cryo-EM map.

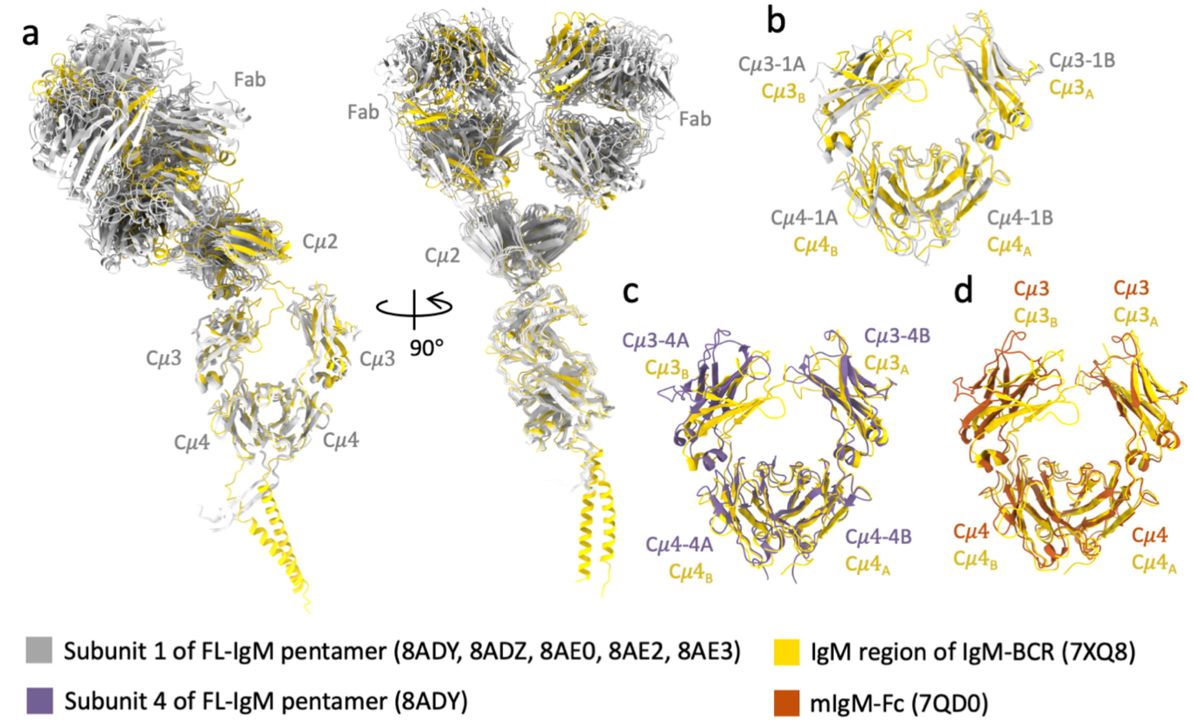

Supplementary Fig. 13. Comparison of Full-length-IgM pentamer and IgM-Fc monomer to IgM-BCR structure. (a) Overlay of subunit 1 in the five conformations of FL-IgM pentamer (in grey) and IgM-BCR (pdb id 7XQ8, in yellow) with Igαβ domains removed, aligned at Cμ4 domains. (b) Overlay of IgM-BCR (Cμ3 and Cμ4 only, in yellow) and asymmetric subunit 1 of IgM pentamer (pdb id 8ADY, Cμ3 and Cμ4 only, in grey) aligned at Cμ4 domains showing similar re-positioning at Cμ3-1A (IgM pentamer) and $C\mu3_B$ (IgM-BCR). (c) Overlay of IgM-BCR (Cμ3 and Cμ4 only, in yellow) and symmetric subunit 4 of IgM pentamer (pdb id 8ADY, Cμ3 and Cμ4 only, in purple) aligned at Cμ4 domains showing mismatch between Cμ3-4A (IgM pentamer) and $C\mu3_B$. (d) Overlay of IgM-BCR (Cμ3 and Cμ4 only, in yellow) and symmetric IgM-Fc monomer (pdb id 7QD0, in brown) aligned at Cμ4 domains, showing superimposed Cμ3 (mIgM-Fc) and $C\mu3_A$ (IgM-BCR). The comparisons of asymmetric subunit 1, symmetric subunit 2-5 of FL-IgM, and IgM-Fc monomer are shown in Fig. 2c and Fig. 5e.

Supplementary Table 1. Data table for predicted and measured FRET and distances in monomeric IgM-Fc

Residue	FRET measured (mean ± SD of fit)	Distance measured (nm)	FRET predicted	Distance predicted (nm)
IgM E526	0.43 ± 0.11 or 0.09 ± 0.06	5.3 (closed) or 7.5 (open)	0.38 (Anti-parallel)	5.4 (Anti-parallel)
			0.84 (Parallel)	3.9 (Parallel)
IgM T556	0.87 ± 0.11 or 0.17 ± 0.10	3.7 (closed) or 6.6 (open)	0.87 (Anti-parallel)	3.7 (Anti-parallel)
			0.55 (Parallel)	4.9 (Parallel)
IgG1 S415	0.50 ± 0.11	5.1	0.40 (Anti-parallel)	5.5 (Anti-parallel)
IgG1 S444	0.90 ± 0.11	3.5	0.95 (Anti-parallel)	3.1 (Anti-parallel)

Supplementary Table 2. Data table for FL-IgM and the five conformations

	FL-IgM	Conformation 1	Conformation 2	Conformation 3	Conformation 4	Conformation 5
**Data Acquisition**						
Voltage	300 kV					
Microscope	FEI Titan Krios					
Camera	Falcon 3, counting					
Calibrated magnification	128,440					
Electron exposure	34.2 e/Å^2					
Exposure rate	0.51 e/Å^2/s					
Number of frames per movie	30					
Automation software	EPU					
Stage tilt	0°					
Defocus range	-1 to -5 μm					
Pixel size	1.09 Å					
**Data processing**						
Data processing packages	Relion, CryoSPARC, CrYOLO					
Initial particle images	1,819,931					
Symmetry imposed	C1	C1	C1	C1	C1	C1
Final particle images	301,286	104,348	50,904	26,368	26,368	16,708
Pixel size Å	1.09	2.18	2.18	2.18	2.18	2.18
Half-map FSC (0.143, masked, Å)	4.3	5.2	6.7	7.1	6.8	8.5
Half-map FSC (0.143, unmasked, Å)	6.0	8.4	9.3	9.4	9.2	12.0
B factor (Å^2)	197	238	328	471	217	504
**Model Refinement**						
Initial model used	6KXS			6KXS, 4JVU, 2AGJ		
Refinement packages	Phenix, Coot					
Map-model FSC (0.5, masked, Å)	6.9	9.4	10.8	10.8	9.1	10.7
Map-model CC						
CC_mask	0.56	0.52	0.45	0.54	0.55	0.52
CC_volume	0.65	0.50	0.43	0.50	0.52	0.47
CC_peaks	0.55	0.49	0.4	0.46	0.49	0.43
CC_box	0.72	0.80	0.77	0.82	0.81	0.81
Model composition						
Non-hydrogen atoms	18,624	27,003	27,003	26,999	26,999	26,999
Protein residues	2371	3455	3455	3454	3454	3454
Ligands	12	16	16	16	16	16

Supplementary Table 3. Data table for mIgM-Fc with C-terminal coiled-coil

	mIgM-Fc
**Data Acquisition**	
Voltage	300 kV
Microscope	FEI Titan Krios
Camera	K2, counting
Calibrated magnification	59,595
Electron exposure	66 e/Å^2
Exposure rate	8.25 e/Å^2/s
Number of frames per movie	40
Energy filter slit width	20 eV
Automation software	EPU
Stage tilt	0°
Defocus range	-1 to -4 μm
Pixel size	0.839 Å
**Data processing**	
Data processing packages	Relion, CryoSPARC, CrYOLO
Initial particle images	7,411,324
Symmetry imposed	C2
Final particle images	961,072
Half-map FSC (0.143, masked, Å)	3.6
Half-map FSC (0.143, unmasked, Å)	3.6
Map sharpening B factor (Å^2)	185
**Model Refinement**	
Initial model used	6KXS
Refinement packages	Phenix, Coot
Map-model FSC (0.5, masked, Å)	3.79
Map-model CC	
CC_mask	0.74
CC_volume	0.71
CC_peaks	0.59
CC_box	0.70
Model composition	
Non-hydrogen atoms	3304
Protein residues	424
Validation	
MolProbity score	1.96
Clashscore	7
Rotamer outliers (%)	0
Ramachandran outliers (%)	0
Cβ outliers (%)	0
Planarity outlier (%)	0
Chirality outlier (%)	0
Bond angle/length outlier (%)	0

Supplementary Table 4. FPS software settings for generation of accessible dye volume clouds

Fluorophore	Link length (nm)	Link width (nm)	Dye radius 1 (nm)	Dye radius 2 (nm)	Dye radius 3 (nm)
Donor (Alexa Fluor 555 C2 maleimide)	2.1	0.45	0.78	0.45	0.15
Acceptor (Alexa Fluor 647 C2 maleimide)	2.1	0.45	1.1	0.47	0.15
