# Supplementary material for: Cryomicroscopy reveals the structural basis for a flexible hinge motion in the immunoglobulin M pentamer
Source: Nat Commun. 2022 Oct 23;13:6314. doi: 10.1038/s41467-022-34090-2 (PMC9588798; doi:10.1038/s41467-022-34090-2)
Supplement: Supplementary file 2 — Description of Additional Supplementary Files [file 41467_2022_34090_MOESM2_ESM.pdf]

File name: Supplementary Movie 1

Description: The hinge motion of $F(ab')_2$ of subunit 1 by 3D variability analysis (3DVA) – front view

File name: Supplementary Movie 2

Description: The hinge motion of $F(ab')_2$ of subunit 1 by 3D variability analysis (3DVA) – side view
