# Supplementary material for: Cryomicroscopy reveals the structural basis for a flexible hinge motion in the immunoglobulin M pentamer
Source: Nat Commun. 2022 Oct 23;13:6314. doi: 10.1038/s41467-022-34090-2 (PMC9588798; doi:10.1038/s41467-022-34090-2)
Supplement: Supplementary file 5 — Reporting Summary [file 41467_2022_34090_MOESM5_ESM.pdf]

nature portfolio

Corresponding author(s): Peter Rosenthal, Pavel Tolar

Last updated by author(s): Oct 6, 2022

Reporting Summary

Nature Portfolio wishes to improve the reproducibility of the work that we publish. This form provides structure for consistency and transparency in reporting. For further information on Nature Portfolio policies, see our Editorial Policies and the Editorial Policy Checklist.

Statistics

For all statistical analyses, confirm that the following items are present in the figure legend, table legend, main text, or Methods section.

n/a	Confirmed	
☐	☒	The exact sample size (*n*) for each experimental group/condition, given as a discrete number and unit of measurement
☒	☐	A statement on whether measurements were taken from distinct samples or whether the same sample was measured repeatedly
☒	☐	The statistical test(s) used AND whether they are one- or two-sided<br>*Only common tests should be described solely by name; describe more complex techniques in the Methods section.*
☒	☐	A description of all covariates tested
☒	☐	A description of any assumptions or corrections, such as tests of normality and adjustment for multiple comparisons
☐	☒	A full description of the statistical parameters including central tendency (e.g. means) or other basic estimates (e.g. regression coefficient) AND variation (e.g. standard deviation) or associated estimates of uncertainty (e.g. confidence intervals)
☒	☐	For null hypothesis testing, the test statistic (e.g. *F*, *t*, *r*) with confidence intervals, effect sizes, degrees of freedom and *P* value noted<br>*Give P values as exact values whenever suitable.*
☒	☐	For Bayesian analysis, information on the choice of priors and Markov chain Monte Carlo settings
☒	☐	For hierarchical and complex designs, identification of the appropriate level for tests and full reporting of outcomes
☒	☐	Estimates of effect sizes (e.g. Cohen's *d*, Pearson's *r*), indicating how they were calculated

Our web collection on statistics for biologists contains articles on many of the points above.

Software and code

Policy information about availability of computer code

Data collection	CryoEM data collected using Thermo Scientific EPU v2.7
Data analysis	CryoEM data processed using following packages: Scipion v2.0, Relion v3.1, CryoSPARC v3.2.0, CTFfind4 v4.1.13, MotionCor2 v1.3.0, crYOLO v1.5.4, PHENIX v1.19.2, UCSF Chimera v1.13.1, Coot v0.9.6, MATLAB 2021b, ImageJ v2.1.0<br><br>Custom MATLAB code to analyse single-molecule FRET from time-lapse images is available at Github (https://github.com/ptolar/single-molecule-FRET-trace-analysis).

For manuscripts utilizing custom algorithms or software that are central to the research but not yet described in published literature, software must be made available to editors and reviewers. We strongly encourage code deposition in a community repository (e.g. GitHub). See the Nature Portfolio guidelines for submitting code & software for further information.

Data

Policy information about availability of data

All manuscripts must include a data availability statement. This statement should provide the following information, where applicable:

- Accession codes, unique identifiers, or web links for publicly available datasets
- A description of any restrictions on data availability
- For clinical datasets or third party data, please ensure that the statement adheres to our policy

The structural data that support the findings of this study have been deposited in the EM Data Bank. The EM maps and models have EMDB/PDB entry numbers EMD-13921, EMD-15375 (pdb id 8ADY), EMD-15376 (pdb id 8ADZ), EMD-15377 (pdb id 8AE0), EMD-15380 (pdb id 8AE3), EMD-15379 (pdb id 8AE2)and EMD-13922 (pdb id 7QDO). Our analysis includes pdb id's 4JVW, 3D03, 6KXS, 4JVU, 2AGJ, 6FCZ, 7XQ8.

Cryo-EM micrographs have been deposited on EMPIAR (id EMPIAR-11077).

Sample data of single-molecule FRET have been provided at Github (https://github.com/ptolar/single-molecule-FRET-trace-analysis).

Field-specific reporting

Please select the one below that is the best fit for your research. If you are not sure, read the appropriate sections before making your selection.

☒ Life sciences ☐ Behavioural & social sciences ☐ Ecological, evolutionary & environmental sciences

For a reference copy of the document with all sections, see nature.com/documents/nr-reporting-summary-flat.pdf

Life sciences study design

All studies must disclose on these points even when the disclosure is negative.

Sample size	All cryoEM datasets consist of several thousand images. The number of images were sufficient to achieve the reported resolution, according to the most commonly reported resolution measure in cryoEM described in Rosenthal and Henderson 2003.
Data exclusions	CryoEM micrographs were excluded if they lacked high-resolution information based on CTF resolution analysis. CryoEM single particle images were excluded after 2D or 3D classifcation as described in the the image processing workflows in the Methods section and in Supplementary Data Figures S1 and S12.
Replication	Structures were determined using independent half datasets, according to standard procedures in cryoEM. Images were collected from three independent replicate prepared grids, which all produced similar images both by low resolution visual inspection and high resolution class averages. There were no unsuccessful replications.
Randomization	Not applicable to this study, as samples were not assigned to experimental groups and data were collected and processed according to standard techniques for cryoEM.
Blinding	Not applicable to this study, as there was no experimental group allocation in data collection and analysis.

Reporting for specific materials, systems and methods

We require information from authors about some types of materials, experimental systems and methods used in many studies. Here, indicate whether each material, system or method listed is relevant to your study. If you are not sure if a list item applies to your research, read the appropriate section before selecting a response.

Materials & experimental systems

n/a	Involved in the study	
☐	☒	Antibodies
☐	☒	Eukaryotic cell lines
☒	☐	Palaeontology and archaeology
☒	☐	Animals and other organisms
☒	☐	Human research participants
☒	☐	Clinical data
☒	☐	Dual use research of concern

Methods

n/a	Involved in the study	
☒	☐	ChIP-seq
☒	☐	Flow cytometry
☒	☐	MRI-based neuroimaging

Antibodies

Antibodies used	anti-human IgM clones HB57 and MU53 (gifts from P. Mongini, Feinstein Institutes for Medical Research) were used with identical results<br>anti-human IgG clone MK1A6 Biorad
Validation	anti-human IgM MU53, HB57 - Rudich, S. M., Mihaesco, E., Winchester, R. & Mongini, P. K. A. Analysis of the domain specificity of various murine anti-human IgM monoclonal antibodies differing in human B lymphocyte signaling activity. Mol Immunol 24, 809–820 (1987).<br>anti -human IgG - Manufacturer validation: ELISA, flow cytometry

Eukaryotic cell lines

Policy information about cell lines

Cell line source(s)	HEK293T - Crick Cell Services

Authentication	HEK293T - STR profiling by Crick Cell Services
Mycoplasma contamination	HEK293T - negative
Commonly misidentified lines (See ICLAC register)	N/A
